# Effects of simulated digestion and prebiotics properties of polysaccharides extracted from *Imperatae Rhizoma* based on different pilot processes

**DOI:** 10.3389/fmicb.2025.1544261

**Published:** 2025-03-07

**Authors:** Mengge Sun, Haotian Huang, Haibao Tang, Jiajie Chen, Wei Chen, Dongsheng Yang

**Affiliations:** ^1^College of Life Science, Zhuhai College of Science and Technology, Zhuhai, China; ^2^College of Life Science, Jilin University, Changchun, China

**Keywords:** IRPs, pilot processes, simulated digestion, short-chain fatty acids, probiotic activity

## Abstract

Recent studies have highlighted the prebiotic potential of natural plant polysaccharides, demonstrating their role in promoting beneficial gut microbiota and improving health. However, research on the digestive properties and prebiotic activities of *Imperatae Rhizoma Polysaccharides* (IRPs) remains limited. This study investigated fresh *Imperatae Rhizoma* as the research object. After processing, dry *Imperatae Rhizoma* and carbonized *Imperatae Rhizoma* were prepared. Three polysaccharides from the fresh, dry, and carbonized *Imperatae Rhizoma* were extracted with traditional hot water. And another polysaccharide was obtained by cold water extraction from fresh *Imperatae Rhizoma*. Total four IRPs were extracted and named: IRPs-F, IRPs-D, IRPs-C, and IRPs-J. This study evaluated the prebiotic activity of four polysaccharides derived from the roots of thatch, demonstrating their resistance to digestion, their ability to promote probiotic growth, and their enhancement of short-chain fatty acid (SCFA) production. The final results show that four IRPs exhibit strong resistance to digestion and IRPs-F ability to promote the growth of beneficial probiotics, making it a promising candidate for functional foods aimed at improving intestinal health, immune regulation, and metabolic benefits. This research is highly relevant to food microbiology and holds significant potential for application in the functional food and gut health sectors.

## 1 Introduction

The *Imperatae Rhizoma* of *Imperata cylindrica* Beauv. var. *major* (Nees) C.E. Hubb., a member of the Gramineae family, which is distributed all over the country. At present, it is widely distributed in the northeast, north, east, south, southwest of China, as well as in Shanxi, Gansu, and other places (Jung and Shin, 2021). It has long been used as a traditional Chinese medicinal herb. Modern research has discovered that *Imperatae Rhizoma* contains various chemical compounds, including polysaccharides, flavonoids, triterpenoids, sterols, organic acids, chromones, and lactones (Fu et al., 2010). Which contribute to its antioxidant, antibacterial, anti-inflammatory, antitumor, liver-protective, kidney-protective and other pharmacological effects (Jiang, 2014; Ruan et al., 2022; Razafindrakoto et al., 2021; Ma et al., 2018). Notably, polysaccharides represent one of the main components of *Imperatae Rhizoma* (Zou et al., 2012) but so far, there has been few studies on *Imperatae Rhizoma* polysaccharides. Yu et al. developed an efficient, economical, and environmentally friendly ultrasound-assisted hot water extraction method for *Imperatae cylindrica* polysaccharides (UICP) and demonstrated that UICP exhibited stronger antioxidant activity and a greater potential to alleviate cell damage than polysaccharide from *Imperatae cylindrica* (ICP) (Yu et al., 2024a). Furthermore, through *in vivo*·fluorescence tracing, *in vitro* simulated digestion, fecal fermentation experiments, and microbial sequencing, demonstrated the digestion and metabolic characteristics of ICP (Yu et al., 2024b). Additionally, ICP was further isolated to significantly improve HK-2 cell injury and apoptosis in hyperuricemic nephropathy mice induced by uric acid stimulation, and reduce uric acid levels (Yu et al., 2023). Pinilla isolated polysaccharides with immunological activity (Pinilla and Luu, 1999).

*Imperatae Rhizoma* has a long history of medicinal use. In the clinical application of traditional Chinese medicine, *Imperatae Rhizoma is* generally divided into three categories: fresh *Imperatae Rhizoma*, dry *Imperatae Rhizoma*, and carbonized *Imperatae Rhizoma* (Gao et al., 2012). However, current research on fresh and carbonized forms is limited, with dried *Imperatae Rhizoma* often used as a reference for clinical applications of fresh *Imperatae Rhizoma*. The existing research also tends to show that the cold and moist nature of fresh *Imperatae Rhizoma* is stronger than that of dry *Imperatae Rhizoma*. Therefore, it is necessary to conduct comparative studies on the functions of different forms of *Imperatae Rhizoma.*

In recent years, people’s health awareness has been increasing, and the link between diet and health has received more and more attention, which has stimulated the demand for functional and healthy food ingredients. As an indigestible food component, prebiotics can be decomposed and utilized by bacteria in the large intestine as a carbon source, selectively stimulate specific gastrointestinal microbiota, promote the growth of beneficial bacteria, and inhibit the growth of harmful bacteria, and promote nutrient absorption and pathogen defense (Gibson et al., 2017; Rau et al., 2024). At the same time, they produce short-chain fatty acids (SCFAs), such as lactic acid, acetic acid, propionic acid, and butyric acid, which are the primary products (Parada Venegas et al., 2019). These SCFAs create an acidic environment in the gut, inhibiting pathogen growth and thereby altering the gut bacterial composition to improve host health (Fusco et al., 2023). It plays a key role in the health of the host. Interestingly, Studies have shown that Mushroom d-glucans (Ruthes et al., 2021), *Lepista sordida* polysaccharides (Wang et al., 2022), and *Flos Sophorae Immaturus* polysaccharides (Zhong et al., 2022) exhibit significant prebiotic potential, aligning with the established definition of prebiotics. This suggests that extracting polysaccharides from natural plants is a convenient and cost-effective approach, with the potential to serve as a promising new source of prebiotics. Due to the absence of polysaccharide-degrading enzymes in the human body, these plant-derived polysaccharides are not absorbed or utilized in the mouth, stomach, and small intestine, categorizing them as indigestible carbohydrates (Kaur et al., 2021). They can selectively promote the growth of beneficial microbes such as bifidobacteria and lactic acid bacteria while enhancing the production of SCFAs, which confer various health benefits to the host.

However, research on the digestive properties and prebiotic activity of IRPs remains scarce, warranting further investigation. Our group extracted and preliminarily purified four kinds of polysaccharides from *Imperatae Rhizoma* and its processed products. Firstly, to simulate the transport of polysaccharide components in the human digestive tract, the experiment was carried out by exposing polysaccharides to simulated oral and gastric conditions, and a simulated human gastrointestinal digestion model was established (Chen et al., 2019). In this model, IRPs were simulated to wriggle under the same conditions of salt solution, pH value, and digestive enzymes in saliva and gastrointestinal tract. The digestibility of artificial gastric juice and α-amylase of the obtained IRPs were evaluated. Finally, 0.5, 1, 1.5, and 2% of four kinds of IRPs were used as carbon sources to replace glucose, and the effects of IRPs on the proliferation of *Lactobacillus acidophilus*, *Lactobacillus casei*, *Lactobacillus plantarum* and *mixed probiotics* in liquid culture were observed. The effects of SCFAs and FOS were compared, and IRPs with optimal prebiotic and functional performance were selected. The selected IRPs and the probiotic strain with the best proliferation effect were further studied, which provided important data for the future prebiotic functional food of IRPs-F in improving obesity, diabetes, and gastrointestinal diseases and regulating the immune system.

## 2 Materials and methods

### 2.1 Materials

Fresh *Imperatae Rhizoma* (purchased from Linyi, Shandong, China), Concentrated sulfuric acid, phenol, and anhydrous ethanol (C_2_H_5_OH, AR) were purchased from Sinopharm Chemical Reagent Co., Ltd. Glucose standards, Coomassie Brilliant Blue, bovine serum albumen (BSA), D(+) glucuronic acid, glucose (AR), fructooligosaccharides, artificial saliva, artificial gastric fluid from Shanghai YuanYe Biotechnology Co., Ltd. DNS reagent MRS medium, and MRS broth (without glucose), (Guangdong Haibo Microbial Technology Co., China). SCFA standards including acetic acid, propionic acid, butyric acid, valeric acid, isobutyric acid, and isovaleric acid were purchased from Aladdin (Shanghai, China). All other chemicals and solvents used are analytical grade (Shanghai, China). *Lactobacillus casei* (*L. casei*; GDMCC 1.410), *Lactobacillus acidophilus* (*L. acidophilus*; GDMCC 1.208) and *Lactobacillus plantarum* (*L. plantarum*; GDMCC 1.1797) (Guangdong Microbial Culture Collection Center, China); *Mixed probiotics* (*M. probiotics*; it is composed of 15 kinds of probiotics, including *Lactobacillus rhamnosus, Bifidobacterium lactis, Lactobacillus salivarius, Bifidobacterium longum, Lactobacillus johnsonii, Bifidobacterium adolescentis, Lactobacillus acidophilus, Lactobacillus paracasei, Lactobacillus reuteri, Streptococcus salivarius, Lactobacillus helveticus, Lactobacillus bulgaricus, Bifidobacterium breve, Bifidobacterium infantis*, and *Lactobacillus plantarum*. Mixed with appropriate amount of glycerol and stored in refrigerator at −80°C, from the laboratory of College of Life Science, Zhuhai College of Science and Technology, Zhuhai, China).

### 2.2 Preparation of IRPs

Select the white, thick fresh *Imperatae Rhizoma*, wash them thoroughly, and cut them into segments. Put a portion of fresh *Imperatae Rhizoma* and dry them in a forced air-drying oven at 70°C for 2–3 days. Record the moisture loss of fresh *Imperatae Rhizoma* before and after drying. Finally, obtain the dry *Imperatae Rhizoma*. The rhizome segments were in a frying pan and the temperature was controlled at 270°C by adjusting the heat of the induction cooker. After 4 min, the quality of the rhizome before and after processing was recorded. Obtained the processed Chinese herb carbonized *Imperatae Rhizoma*.

### 2.3 Extraction and purification of IRPs

Weighed fresh *Imperatae Rhizoma*, dry *Imperatae Rhizoma* and carbonized *Imperatae Rhizoma* then placed them in a round-bottom flask, using water as the extraction solvent. Traditional hot-water extraction parameters were as follows: extraction time of 1.5 h, temperature was controlled at 100°C, and material-to-liquid ratio of 1:6 (g/mL) (Lu et al., 2021). This process was repeated three times.

Weighed a certain quantity of fresh *Imperatae Rhizoma*, added cold water at a material-to-liquid ratio of 1:6 (g/mL). The cold-water extraction method was then employed by allowing the mixture to stand at 4°C in a refrigerator for 12 h.

Finally, three different polysaccharides were extracted from hot-water: fresh *Imperatae Rhizoma* polysaccharides (IRPs-F), dry *Imperatae Rhizoma* polysaccharides (IRPs-D), and carbonized *Imperatae Rhizoma* polysaccharide (IRPs-C) and one polysaccharide from cold-water extraction and named *Imperatae Rhizoma* juice (IRPs-J). After the extraction, the supernatants were combined, centrifuged, and filtered, then concentrated to 1/4 volume approximately. Four polysaccharide solutions were precipitated overnight with 80% ethanol, and the bottom precipitate obtained by centrifugation was the polysaccharides. The precipitate of four IRPs was redissolved with an appropriate volume of water. And selected the Sevage reagent to remove free proteins from IRPs (Huang et al., 2014). Then the solution was concentrated to remove the residual organic reagent and dialyzed against water for 12 h. Added Fourfold volumes of ethanol to the solution again, collect the precipitate and wash it with ethanol to remove impurities and finally lyophilized. IRPs-F, IRPs-D, IRPs-C, IRPs-J were obtained, respectively.

### 2.4 Chemical compositions

The total sugar content was determined by the phenol-sulfuric acid method with glucose as the equivalents (Ren et al., 2019). The uronic acid content was determined by the carbazole-sulfuric method, and glucuronic acid was used as the standard (Zhou et al., 2018). Protein content was determined with Kemas Brilliant Blue assay using bovine serum albumin for standards (Zou et al., 2023). The polyphenol content was calculated with gallic acid as the standard (Nguyen Thi et al., 2021).

### 2.5 Determination of UV

The above four IRPs solution was prepared as 0.5 mg/mL, and scanned by ultraviolet spectroscopy (Shimadzu, Japan) in the range of 200–400 nm (Liu et al., 2022).

### 2.6 Determination of Fourier transform infrared spectroscopy

The completely dried KBr and four IRPs were thoroughly mixed and ground in an onyx mortar. After pressing into pellets, the infrared spectrum was scanned in the wavenumber range of 400 cm^−1^ to 4,000 cm^−1^ (Liang et al., 2023).

### 2.7 *In vitro* simulated digestion of polysaccharides

#### 2.7.1 Determination of reducing sugar

The content of reducing sugar in IRPs was determined by the DNS method with glucose as the standard (Xu et al., 2018). The reaction mixture that contained different concentrations of glucose and DNS reagent was mixed with shaking adequately. Then, place the mixture in the water at 90°C and allow it to react thoroughly for 5 min, taken out and placed in an ice bath to room temperature. The reaction solution was added with water to a final volume of 10 mL. The absorbance value at 540 nm was measured. Drawn the standard curve and calculated the regression equation *y* = 1.5994*x* − 0.0014 with associated coefficient *R*^2^ = 0.9991.

#### 2.7.2 Resistance to artificial saliva digestion

The final pH of artificial saliva was adjusted to 4, 5, 6, 7, and 8 using the concentration of HCl and NaOH solution is 0.1 mol/L. Take the IRPs (10 mg / mL) and the positive control group FOS solution were mixed with artificial saliva of different pH in equal volumes (Ma et al., 2019). After mixing, place in a 37°C, 110 r/min oscillator for constant temperature incubation for 6 h. The reaction mixtures were collected at 0, 0.5, 1, 2, 4, and 6 h, respectively. Then boil in a water bath for 10 min to inactivate the enzyme. Measured the reducing sugar and total sugar contents, and calculated the degree of hydrolysis of IRPs. Each experiment was repeated three times.

The calculation equation of polysaccharide hydrolysis degree is as follows:

$$\mathrm{hydrolysis degree}\;\left(\%\right)=\frac{\mathrm{reducing sugar increased}}{\begin{array}{l} \mathrm{total sugar}-\mathrm{primary}\;\\ \mathrm{reducing sugar} \end{array}}\times 100\%$$

Here, the amount of reduced sugar released was calculated as the difference between the reduced sugar content and the initial reduced sugar content.

#### 2.7.3 Resistance to artificial gastric and intestinal juice digestion

Adjust the pH of the artificial gastric juice to 1, 2, 3, 4, and 5. Then, mix four IRPs with equal volumes of artificial gastric juice, and incubate the mixture at 37°C. The reaction mixtures simulating gastric digestion were collected at 0, 0.5, 1, 2, 4, and 6 h (Dong et al., 2020). After incubation, the mixtures were boiled in a water bath for 10 min to inactivate the enzymes. The degree of hydrolysis of the IRPs was calculated as described for the artificial saliva digestion. FOS served as the positive control, and each experiment was repeated three times.

### 2.8 Prebiotic activity of polysaccharides

The MRS culture was sterilized at 121°C for 20 min, then freeze-dried powder of *L. acidophilus, L. casei*, *L. plantarum*, and *M. probiotics* were inoculated into 50 mL sterile MRS medium, respectively. The strains were cultured in a shaker (SKY-2112B, Shanghai Huyueming Scientific Instrument Co., Ltd., China) at 37°C, 110 r/min for 24 h, and then transferred three times to activate the strains. The activated strains using a McClatchy turbidimeter and sterile sugar-free MRS medium were diluted to the final concentration of 10^8^ CFU / mL (Wang et al., 2022).

#### 2.8.1 Prebiotic activity of IRPs

The study investigated the *in vitro* prebiotic activity of IRPs, using no additional carbon source MRS medium as a blank control and the recognized prebiotic FOS as a positive control. Four IRPs and FOS were added to the sugar-free MRS broth and then configured into the medium with concentrations of 0, 0.5, 1.0, 1.5, and 2.0% (w/v) (Chen et al., 2019). After sterilization at 121°C for 20 min, inoculation of 5% diluted bacterial suspensions of four probiotics, suspension and mixed well. All samples were incubated at 37°C for 48 h. The optical density of probiotics in each group was measured at 600 nm, and the experiment was repeated three times.

#### 2.8.2 The growth curves of different IRPs on probiotics

The bacterial growth curve analysis was performed according to the method of Liang et al., and was modified to determine the effects of four IRPs on the growth curves of *L. acidophilus*, *L. plantarum*, *L. casei*, and *M. probiotics* (Liang et al., 2023) 0.200 μL of MRS medium containing 2% of four IRPs was added to the sterile 96-well plate, the MRS medium containing FOS was used as the positive control, and the MRS medium without sugar was used as the blank control. Each well was inoculated with 10 μL of *L. casei, L. plantarum, L. acidophilus*, and *M. probiotics* solutions (108 CFU / mL). After fully mixed, the 96-well plates were cultured in the constant temperature air bath shaker at 37°C, 100 rpm. The OD value at 600 nm was measured and recorded every 3 h, and the growth curve of probiotics was drawn by analyzing the experimental data. Each group of experiments was operated three times. Screen out the IRPs and strains as the best carbon and microbial strains, and further analyze their growth and metabolism processes.

The obtained data finally adopted the Logistic model in Origin 2021 software, and the iterative algorithm was used to fit the growth curve with the Levemberg-Marquardt optimization algorithm (Crispim et al., 2015). Finally, the fitting growth curves of four probiotics were obtained. The specific expression of the algorithm is as follows:

$$y=A_{2}+\left(A_{1}-A_{2}\right)/{\left(1+\left(X/X_{0}\right)\right)}^{p}$$

Among them: *A*_1_, *A*_2_, *X*_0_, and *p* are parameters. *A*_1_ represents the degree of deviation between the true curve and the model; *A*_2_ is the maximum growth value predicted by the model; *X*_0_ is the inflection point time when the growth rate of probiotics reaches the maximum; the curve has the maximum slope at the crossing point (*X*_0_, *A*_2_), that is, the maximum growth rate; p denotes the growth rate coefficient.

#### 2.8.3 The analysis of medium SCFAs

SCFAs were determined using a recorded gas chromatography (GC-2014, Shimadzu Corporation, Kyoto, Japan) procedure with minor modifications (Nie et al., 2020). To collect SCFAs for analysis, the fermentation broth was centrifuged at 8,000 r/min for 5 min. The supernatant was taken and 100 μL of 50% sulfuric acid was added for acidification. After shaking and mixing, an equal volume of ethyl acetate was added. The mixture was fully mixed with a vortex mixer for about 1 min to completely dissolve the SCFAs in the sample to be tested (Mao et al., 2023). Specifically, it was carried out on a GC-2014 gas chromatography system equipped with a Rtx-Wax fused silica capillary column (30 m × 0.25 mm × 0.25 μm), the flame ionization detector (FID) and the HS-20 headspace sampling device. The operating conditions were as follows: split ratio: 1: 10; FID detector temperature: 230°C; The temperature program was as follows: the initial temperature was 80°C, increased to 180°C at the rate of 10°C/min, and then increased to 220°C at a rate of 20°C/min. The total analysis time was 12 min, and the flow rates of hydrogen, air, and nitrogen were 30 mL/min, 300 mL/min, and 19.3 mL/min, respectively. HS-20 headspace sampling conditions were as follows: constant temperature furnace temperature 70°C, sample flow path temperature 100°C, transmission line temperature 150°C, sample bottle pressure time 1 min.

#### 2.8.4 Analysis of medium pH, total sugar, and lactic acid

The *L. plantarum* suspension was inoculated into the optimal carbohydrate source and cultured at intervals of 0, 6, 12, 18, 24, 30, and 36 h. After centrifugation at 8,000 rpm for 5 min, the supernatant was collected and stored at −20°C for further analysis. The pH of the fermentation broth was measured using a pH meter. Lactic acid content was quantified using the p-hydroxyphenyl method, while total sugar content was assessed by the phenol-sulfuric acid method (Zhong et al., 2022).

### 2.9 Statistical analysis

All experiments were performed three times. Data were expressed as mean ± standard deviation (SD). To compare the differences between multiple groups, the data were analyzed by ANOVA. *p* < 0.05 was considered statistically significant. Origin 2021 and IBM SPSS Statistics 26 were used for statistical analysis.

## 3 Results

### 3.1 Analysis of chemical compositions

**Figure 1:**
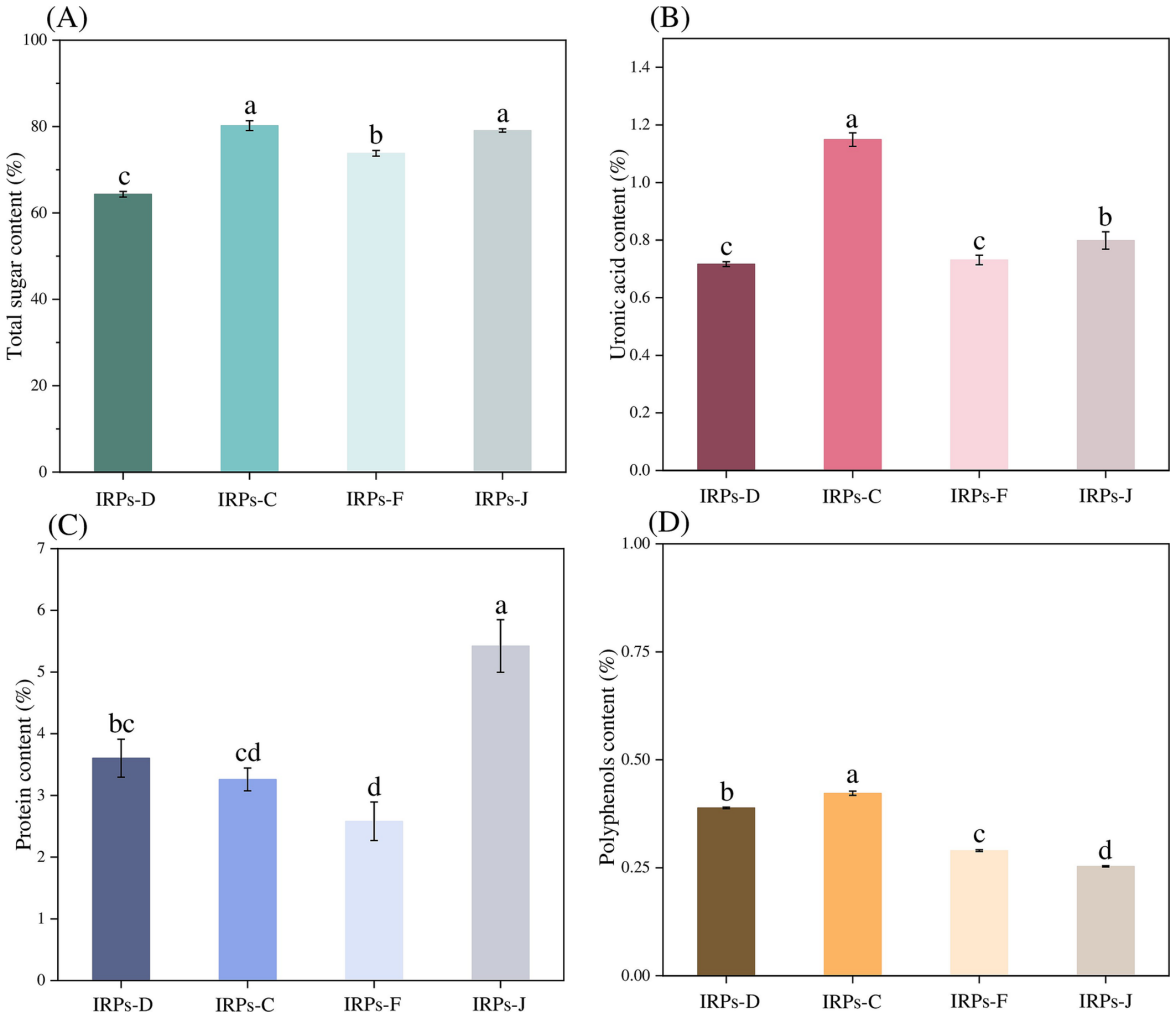
The total sugar contents **(A)**, uronic acid content **(B)**, protein content **(C)**, and polyphenols content **(D)** of four different IRPs. Different lowercase letters indicate significant differences for *p* < 0.05.

The phenol-sulfuric acid method was employed to construct a standard calibration curve, with absorbance values plotted on the ordinate and the glucose concentrations on the abscissa. The resulting standard curve was described by the equation *y* = 7.4355 + 0.1456, with a correlation coefficient *R*^2^ = 0.9995, indicating a strong linear relationship. Based on this standard curve, as shown in Figure 1A, the results showed that the total sugar contents of IRPs-D, IRPs-C, IRPs-F, and IRPs-J were calculated to be 64.34 ± 0.648%, 80.21 ± 1.147%, 73.80 ± 0.659%, and 79.08 ± 0.377%, respectively.

Numerous studies have demonstrated that the content of uronic acids in polysaccharides is closely related to their biological activity (Shang et al., 2019; Li et al., 2022). Therefore, in this study, the uronic acid content of four different types of polysaccharides from *Imperatae Rhizoma* was measured using the sulfuric acid-carbazole method. A standard calibration curve was constructed by plotting absorbance values on the ordinate and the concentration of the standard on the abscissa. The resulting standard curve was described by the equation *y* = 22.729*x* + 0.1938, with a correlation coefficient *R*^2^ = 0.9993, indicating a good linear relationship. As shown in Figure 1B, the uronic acid contents of IRPs-D, IRPs-C, IRPs-F, and IRPs-J were determined to be 0.72 ± 0.008%, 1.15 ± 0.024%, 0.73 ± 0.016%, and 0.80 ± 0.030%, respectively. The low uronic acid content in all four IRPs suggests that they were neutral polysaccharides.

Although the Sevage method was used to remove proteins from the IRPs, trace amounts of protein may still remain in the samples. To determine the protein content, the Coomassie Brilliant Blue G-250 method was employed, with absorbance values plotted on the ordinate and the concentration of bovine serum albumin standard on the abscissa. The resulting standard curve was described by the equation *y* = 3.8354*x* + 0.6258, with a correlation coefficient *R*^2^ = 0.9994, indicating a good linear relationship. As observed from Figure 1C, the protein contents of IRPs-D, IRPs-C, IRPs-F, and IRPs-J were calculated to be 3.60 ± 0.308%, 3.26 ± 0.184%, 2.58 ± 0.312%, and 5.42 ± 0.427%, respectively. To further investigate the biological activity of these polysaccharides, additional purification steps, such as cellulose column chromatography, are necessary to eliminate the potential effects of residual proteins.

Finally, the polyphenols content was determined using the Folin–Ciocalteu method. The standard calibration curve was constructed by plotting absorbance values on the ordinate and gallic acid concentrations on the abscissa. The resulting standard curve was described by the equation *y* = 65.052*x* + 0.0191, with a correlation coefficient *R*^2^ = 0.9992, indicating a strong linear relationship. It can be observed from Figure 1D that the polyphenolic contents of IRPs-D, IRPs-C, IRPs-F, and IRPs-J were determined to be 0.39 ± 0.002%, 0.42 ± 0.005%, 0.29 ± 0.002%, and 0.25 ± 0.001%, respectively.

### 3.2 Analysis of UV spectroscopy

**Figure 2:**
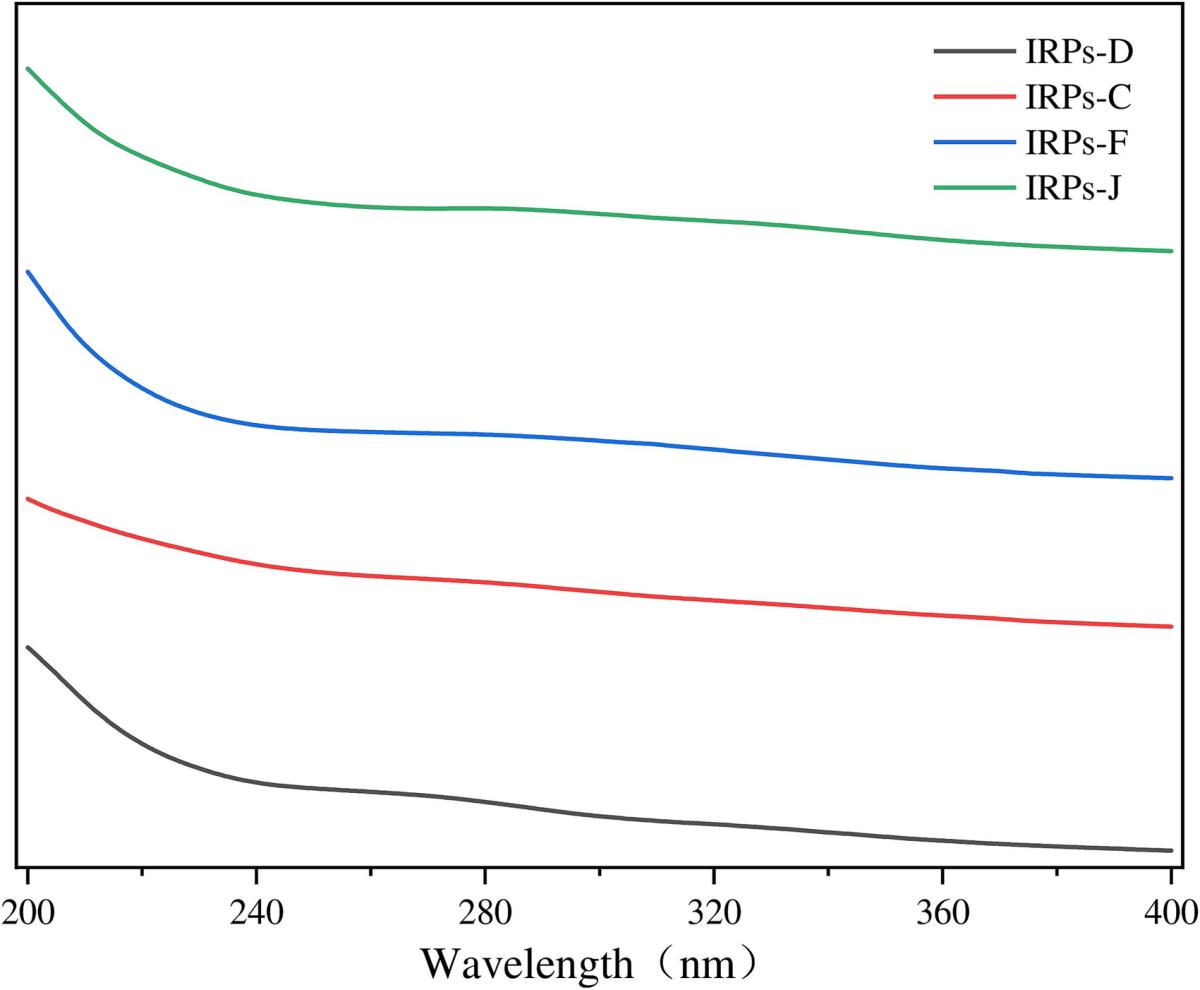
The results of Ultraviolet spectra of different components of IRPs.

Nucleic acids and proteins exhibit characteristic UV absorption peaks at 260 and 280 nm, respectively (Zhao et al., 2022). Therefore, performing UV spectrum scanning on polysaccharides can accurately identify the presence of proteins and nucleic acids within the polysaccharides. The UV absorption spectra of the four IRPs within the wavelength range of 200–400 nm were shown in Figure 2. The results indicate that the four IRPs exhibit no significant absorption peaks at 260 and 280 nm, demonstrating that the four IRPs contain minimal amounts of nucleic acids and proteins, and that the preliminary purification process was effective.

### 3.3 Analysis of FT-IR spectroscopy

**Figure 3:**
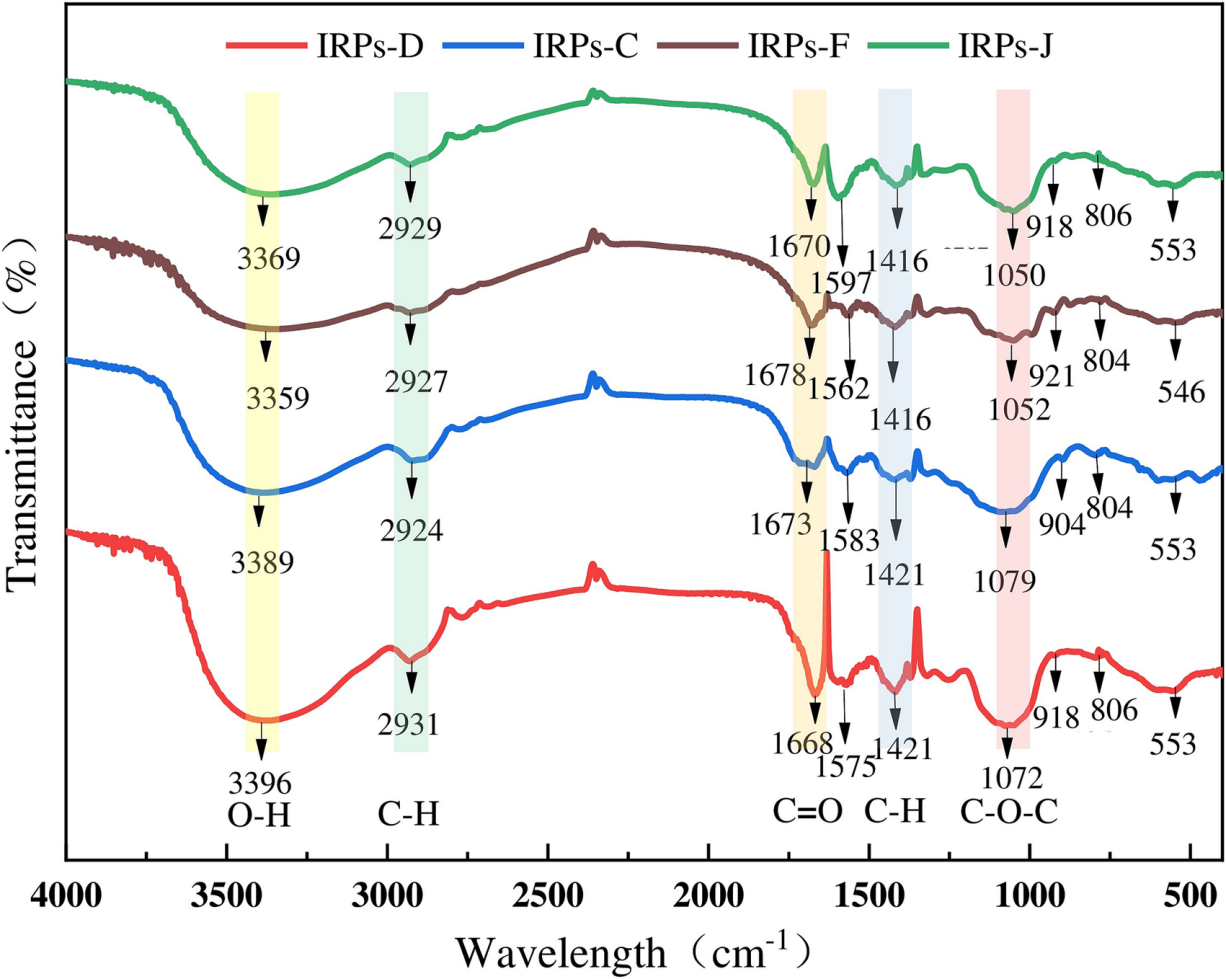
The results of FT-IR spectrograms of four different IRPs.

The IR spectrum scanning results of four different IRPs are shown in Figure 3. The results indicate that IRPs have the characteristic absorption peaks of polysaccharides. Specifically, a strong absorption peak was observed near 3,300 cm^−1^, attributed to the stretching vibration of O-H bonds. A sharp weak absorption peak appears near 2,900 cm^−1^, which is formed by C-H stretching vibration (including methyl − CH_3_, methylene − CH_2_, methylene − CH), and commonly appears in the hydrocarbon skeleton of polysaccharides. Similarly, the absorption peak at about 1,670 cm ^−1^ is attributed to the N-H angular vibration of the amino group of the protein species, the C=O symmetric stretching vibration of the carboxyl group, or the bound water peak in the polysaccharides. Peaks in the vicinity of 1,550–1,600 cm^−1^ and 1,400 cm^−1^ correspond to the protonated carboxyl C=O antisymmetric stretching and C-H bending vibrations. Notably, IRPs-D and IRPs-F show distinct peaks around 1,458 cm^−1^, suggesting a higher presence of CH_2_ and CH_3_ groups in these polysaccharides. Absorption peaks within the range of 1,200–1,300 cm^−1^ are attributed to the asymmetric vibrations of C-O-C or C-OH on the sugar ring, it is a common resonance absorption peak of the pyranose ring lactone and hydroxyl group, which is the characteristic absorption peak of pyranose. It has been confirmed that the sugar residues of the four IRPs exist in the form of pyranose. The absorption peak around 920 cm^−1^ is due to asymmetric ring stretching vibrations of the pyranose ring, indicating that the polysaccharide structure contains β-glycosidic bonds, while the weak absorption peak at 800 cm^−1^ corresponds to α-glycosidic bonds. The absorption peak near 590 cm^−1^ is formed by the out-of-plane bending vibration of −OH groups.

In summary, the infrared spectra of these four polysaccharides demonstrate similar structural characteristics, including characteristic peaks of O-H, C-H, C=O, and sugar rings (Shen et al., 2023). The four IRPs are identified as poly-pyranoses with essentially the same carbon chain skeleton, incorporating both α and β configuration glycosidic linkages, probably. However, there are slight differences in the absorption intensity of some bands, which may be attributed to variations in their molecular structures and functional group compositions.

### 3.4 Analysis of vitro simulated digestion

#### 3.4.1 Digestibility of four IRPs by artificial saliva

**Figure 4:**
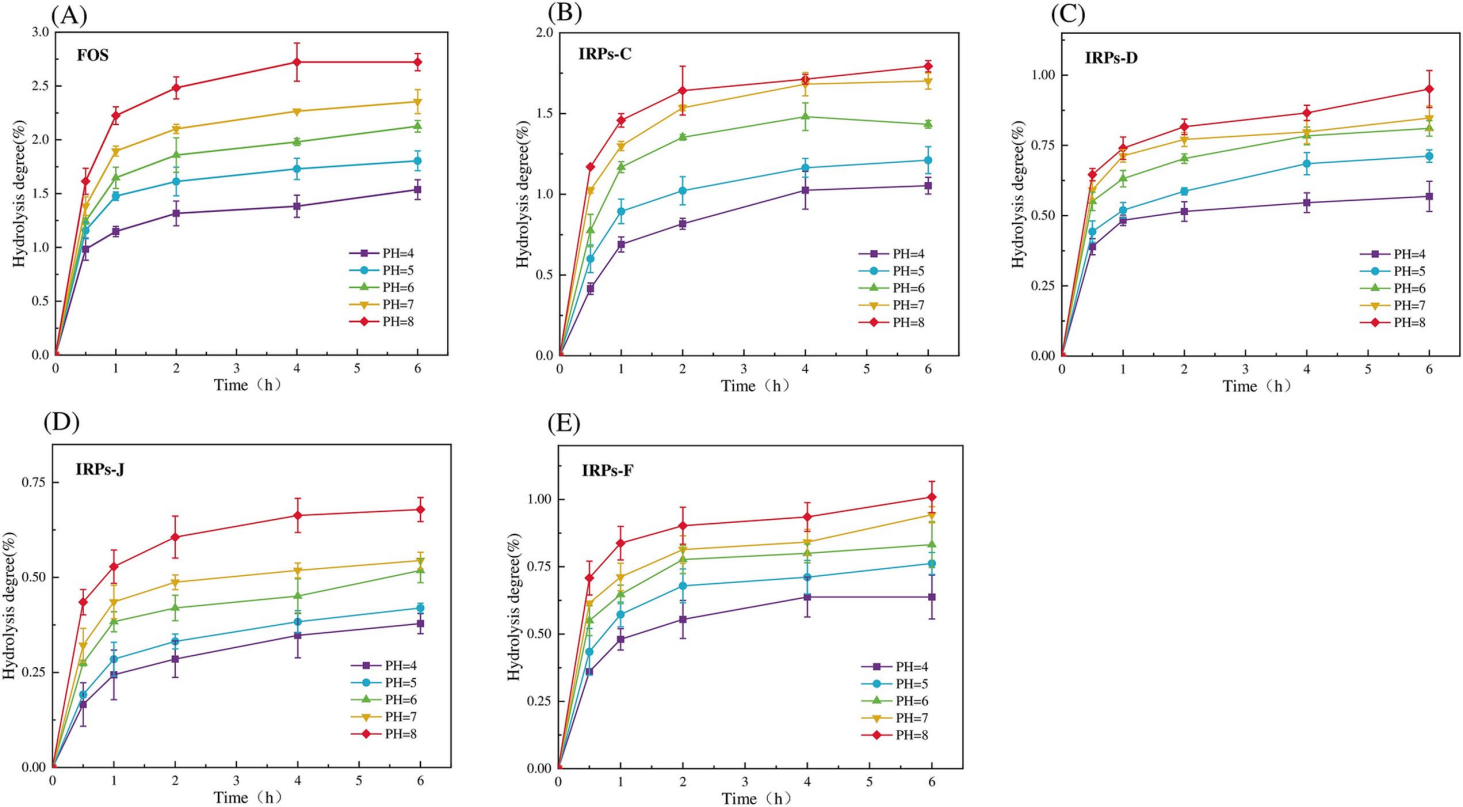
Resistance of FOS and the four IRPs fractions to artificial saliva. **(A)** represents FOS, **(B)** represents IRPs-D, **(C)** represents IRPs-C, **(D)** represents IRPs-F, and **(E)** represents IRPs-J.

Evaluating the anti-hydrolysis ability of polysaccharides is a key criterion for judging whether polysaccharides can be used as prebiotics. Only polysaccharides with anti-digestibility can reach the intestine smoothly without being digested, for the decomposition and utilization of probiotics in the intestine, and play the role of their specific prebiotics (Peng et al., 2024). To evaluate whether different IRPs meet this standard, the hydrolysis resistance of four IRPs to artificial saliva was studied. As shown in Figures 4A–E represent the degree of hydrolysis of FOS, IRPs-D, IRPs-C, IRPs-F, and IRPs-J in artificial saliva, respectively. The results indicated that both hydrolysis time and pH significantly influenced the hydrolysis degree of IRPs. Specifically, as the pH increased, the degradation degree followed the trend: 8 > 7 > 6 > 5 > 4. With the prolongation of incubation time, the degree of hydrolysis of polysaccharides increased rapidly at first, then tended to be gentle, and the degree of hydrolysis gradually stabilized at 4–6 h. After incubation at pH = 8 for 6 h, the maximum degree of hydrolysis of IRPs-D, IRPs-C, IRPs-F, and IRPs-J was 0.95 ± 0.07%, 1.79 ± 0.04%, 1.01 ± 0.06% and 0.68 ± 0.03%, respectively, which were significantly lower than 2.72 ± 0.08% of FOS. These findings suggest that the four IRPs exhibit strong resistance to hydrolysis in artificial saliva containing α-amylase. Among them, the anti-hydrolysis ability ranked as IRPs-J > IRPs-F ≈ IRPs-D > IRPs-C, which may be influenced by differences in extraction or processing methods.

#### 3.4.2 Digestibility of four IRPs in artificial gastric and intestinal juice

**Figure 5:**
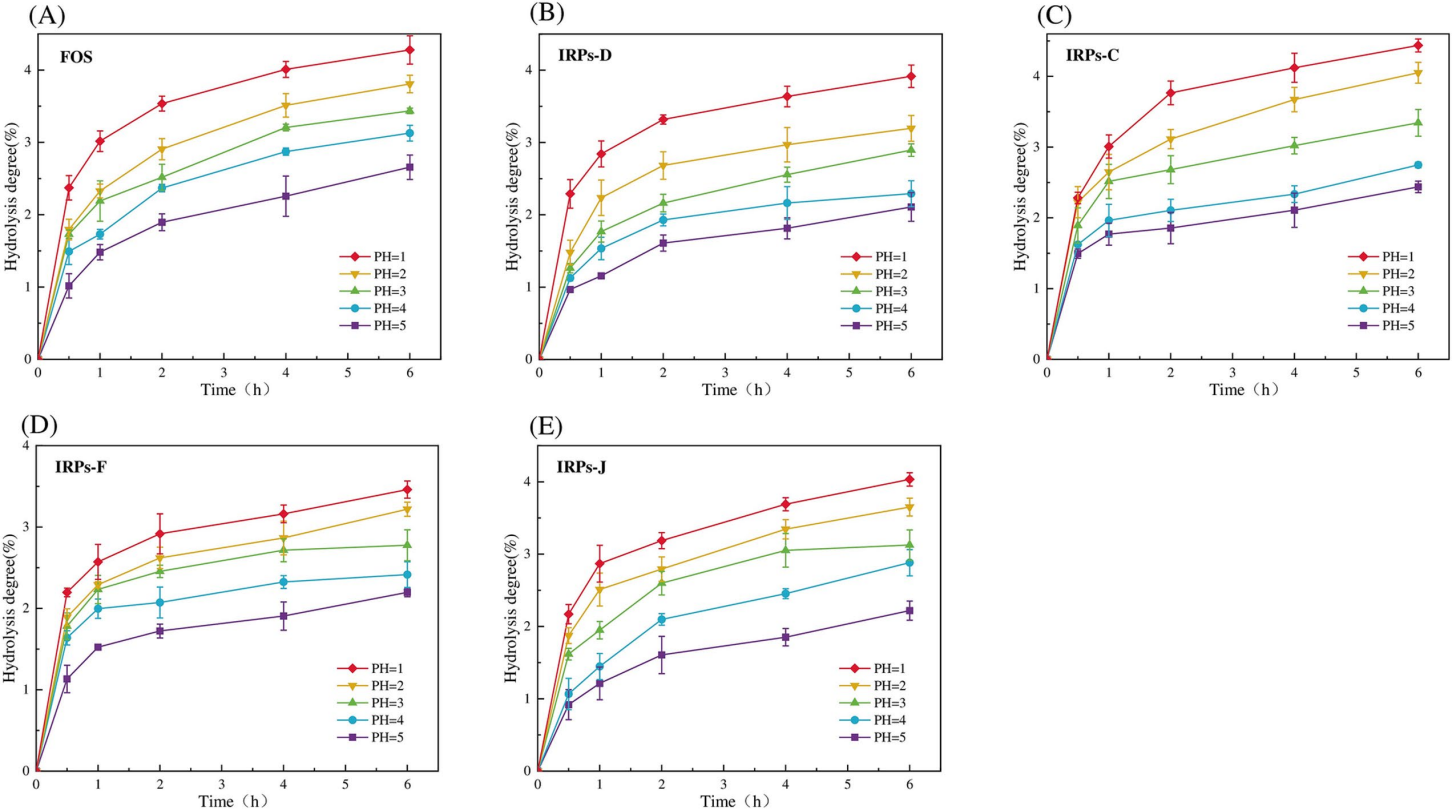
Resistance of FOS and four different IRPs fractions to artificial gastric juice. **(A)** represents FOS, **(B)** represents IRPs-D, **(C)** represents IRPs-C, **(D)** represents IRPs-F, and **(E)** represents IRPs-J.

In this study, the anti-hydrolysis ability of different IRPs in simulated gastric juice was systematically analyzed by *in vitro* simulated digestion experiments. As much as possible to simulate the human gastrointestinal digestion environment including acidic conditions of gastric juice (pH 1–5), simulated gastric emptying time (4–6 h), simulated gastric digestion peristalsis (rotation speed 110 rpm), and human body temperature (37°C). Figures 5A–E shows the results of the degree of hydrolysis of FOS, IRPs-D, IRPs-C, IRPs-F, and IRPs-J in artificial gastric juice with different pH values over time. The results showed that the degree of hydrolysis of different polysaccharides in gastric juice increased significantly with time, and the lower the pH value, the higher the degree of hydrolysis. Therefore, the hydrolysis degree of IRPs-D, IRPs-C, IRPs-F, and IRPs-J reached the maximum value of 3.92 ± 0.15%, 4.44 ± 0.09%, 3.46 ± 0.10% and 4.03 ± 0.09%, respectively, after 6 h hydrolysis in artificial gastric juice with pH = 1. Compared with the positive control group FOS, the maximum hydrolysis degree of the four polysaccharides was less than 5%, and they still maintained good anti-hydrolysis characteristics, indicating that they had good stability in an acidic environment. The anti-hydrolysis ability of the four IRPs was IRPs-F > IRPs-D > IRPs-J > IRPs-C, which may be caused by many factors, such as molecular weight, monosaccharide composition (pyranose, furanose, uronic acid content), branching degree of sugar chain, helix structure, head conformation, glycosidic bond type, and connection mode. These results provide a theoretical basis for IRPs as potential prebiotics.

In summary, similar to the anti-digestive ability of *acanthopanax senticosus* polysaccharide (Wang et al., 2024), mallotus oblongfolius polysaccharides (Tan et al., 2024) and litch polysaccharides (He et al., 2022), four IRPs also have good anti-digestive ability in artificial gastric juice, and more than 95% of the polysaccharides are not degraded, indicating that these four IRPs components can resist the degradation of gastric acid and digestive enzymes, can reach the intestinal tract safely, and reach the intestinal tract in a complete form for the use of probiotics in the intestinal tract, which meets the primary standard of prebiotics.

### 3.5 Screening for optimal concentrations of IRPs to promote probiotic growth

**Figure 6:**
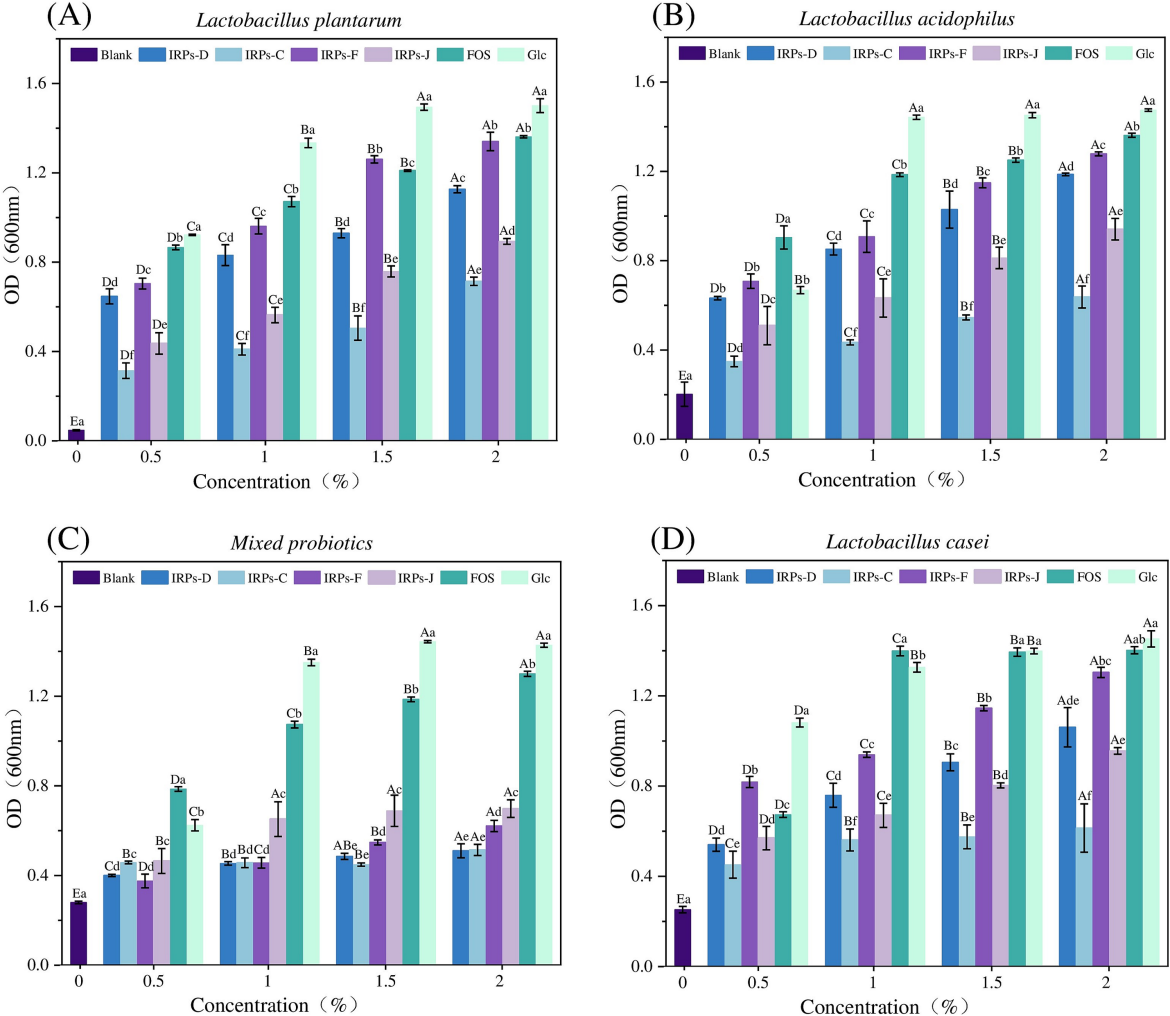
Effects of Glc FOS and four IRPs on the proliferation of Lactobacillus strain after 48 h incubation. *Lactobacillus plantarum* **(A)**, *Lactobacillus acidophilus* **(B)**, *Lactobacillus casei* **(C)**, and *Mixed probiotics* **(D)**.

Probiotics have significant benefits for human health, including the inhibition of pathogen growth through competition in the intestine, as well as the prevention and alleviation of intestinal diseases such as diarrhea, constipation, and irritable bowel syndrome, by enhancing immune system function (Yang et al., 2023). Probiotics also play a crucial role in the development of the intestinal mucosal immune system. Long-term probiotic intake helps maintain the intestinal barrier function and reduces the risk of excessive immune responses. Moreover, probiotics are widely used in the food industry, particularly in yogurt, fermented foods, functional beverages, and other products. To investigate whether IRPs can be metabolized and utilized by probiotics in the intestine, we studied the effects of different IRP components on probiotic growth *in vitro*. IRPs were added to sugar-free MRS basal medium as the sole carbon source, with FOS and glucose (Glc) as positive controls, to compare their growth-stimulating effects on *L. acidophilus*, *L. casei*, *L. plantarum*, and *M. probiotics* culture composed of 15 different strains. Figure 6 illustrates the impact of various polysaccharide concentrations (0.5, 1, 1.5, and 2%) on the proliferation of the four probiotics.

The results showed that the positive control monosaccharide Glc, had the most significant proliferative effect on four probiotics, as Glc directly provides energy for growth via a relatively simple metabolic pathway. FOS also significantly promoted probiotic growth, providing energy for the bacteria, though its metabolic efficiency is slightly lower than glucose. Both FOS and Glc significantly promoted the growth of probiotics as the sole carbon sources.

As shown in Figure 6A, for *L. plantarum,* four IRPs promoted bacterial growth, exhibiting a concentration-dependent proliferation effect, with bacterial numbers increasing from 0.5 to 2.0%. The maximum proliferation was observed at 2.0% IRPs concentration. At this concentration, IRPs-F had a proliferation effect similar to that of FOS, and significantly higher than IRPs-D, IRPs-J, and IRPs-C. As shown in Figure 6B, for *L. acidophilus,* all IRPs stimulated growth, and polysaccharide concentrations between 0.5 and 2.0% exhibited a concentration-dependent proliferative effect. At 2%, IRPs-F demonstrated the best growth-promoting effect, although it was significantly lower than FOS. Similarly, As shown in Figure 6C, for *L. casei,* the effect of IRPs on growth increased with concentration from 0.5 to 2.0%, with four IRPs showing the best growth-promoting effect at 2.0%. Figure 6D shows the effect of different IRPs concentrations on *M. probiotics* growth, which increased in a dose-dependent manner from 0.5 to 2.0%.

In general, proliferation across all carbon sources showed a trend of increasing final probiotic concentration with higher carbon source concentrations. The order of prebiotic activity was IRPs-F > IRPs-D > IRPs-J > IRPs-C. At a concentration of 2.0%, IRPs-F had the most significant proliferation effect on *L. plantarum,* which was not significantly different from FOS. Thus, 2.0% IRPs was selected as the optimal carbon source concentration to further investigate growth rate indicators in probiotics under different carbon sources.

### 3.6 Growth curve

**Figure 7:**
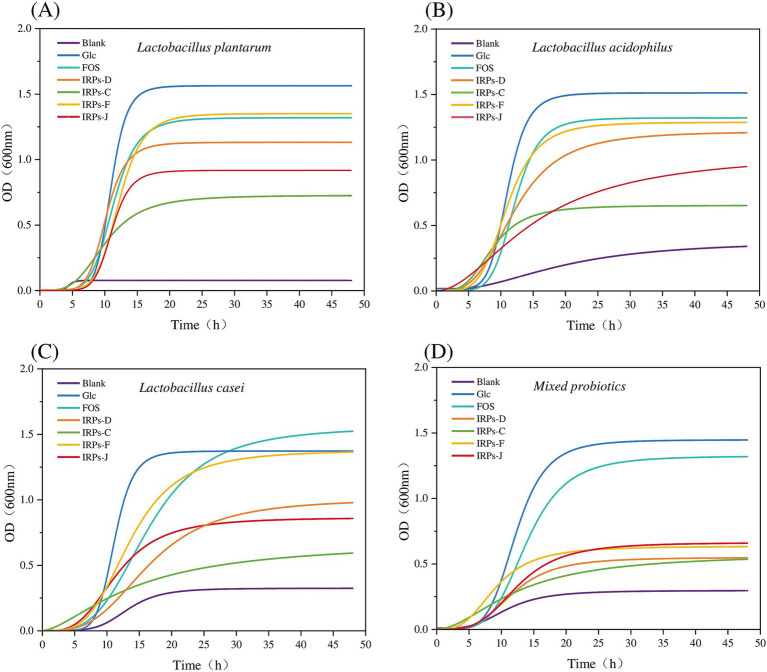
Growth curves of *Lactobacillus plantarum* **(A)**, *Lactobacillus acidophilus* **(B)**, *Lactobacillus casei* **(C)**, and *Mixed probiotics* **(D)** after treatment with four different IRPs.

Under the condition of adding 2% IRPs as a carbon source to the sugar-free MRS basal medium, the growth curves of four probiotics, with various IRP components, as well as the positive controls Glc and FOS, are shown in Figure 7, and the fitting parameters are listed in Table 1. The coefficient of determination (R^2^) for model fitting was greater than 0.9, indicating a good fit. Analysis of Figure 7A revealed that *L. plantarum* had a short lag phase (0–6 h) under all carbon source conditions, suggesting strong adaptability to IRPs and its ability to utilize the four IRPs components. In contrast, Figure 7B shows that *L. acidophilus* exhibited slower growth with IRPs-C or IRPs-J, indicating lower utilization efficiency for these carbon sources. Figures 7C,D show that the lag phase for *L. casei* and *M. probiotics* with IRPs was slightly longer than that for the positive controls, Glc and FOS.

**Table 1:** Growth kinetics equation nonlinear fitting parameters.

Bacteria	Carbon source	*A*_1_	*A*_2_	*X*_0_	*p*	*R* ^2^
*L. plantarum*	Blank	0.0010 ± 0.008	0.0775 ± 0.008	4.4392 ± 1.526	9.9729 ± 31.696	0.8103
*L. plantarum*	Glc	0.0019 ± 0.003	1.5638 ± 0.021	10.9427 ± 0.261	9.0160 ± 1.053	0.9987
*L. plantarum*	FOS	0.0041 ± 0.006	1.3198 ± 0.019	11.3484 ± 0.256	6.1871 ± 0.578	0.9982
*L. plantarum*	IRPs-D	0.0014 ± 0.003	1.1319 ± 0.013	10.2045 ± 0.434	6.7553 ± 1.306	0.9988
*L. plantarum*	IRPs-C	0.0011 ± 0.001	0.7275 ± 0.011	10.0886 ± 0.481	3.6202 ± 0.403	0.9987
*L. plantarum*	IRPs-F	0.0033 ± 0.004	1.3513 ± 0.015	12.1811 ± 0.107	6.6279 ± 0.389	0.9993
*L. plantarum*	IRPs-J	0.0019 ± 0.005	0.9177 ± 0.035	11.1006 ± 0.457	7.8748 ± 1.586	0.9940
*L. acidophilus*	Blank	0.0026 ± 0.003	0.3820 ± 0.088	19.3531 ± 4.113	2.3102 ± 0.317	0.9742
*L. acidophilus*	Glc	0.0188 ± 0.004	1.5120 ± 0.019	11.0433 ± 0.283	7.2006 ± 0.948	0.9986
*L. acidophilus*	FOS	0.0022 ± 0.003	1.3214 ± 0.015	12.1154 ± 0.274	6.5111 ± 0.478	0.9990
*L. acidophilus*	IRPs-D	0.0021 ± 0.006	1.2189 ± 0.031	12.0887 ± 0.909	3.4356 ± 0.354	0.9980
*L. acidophilus*	IRPs-C	0.0010 ± 0.001	0.6533 ± 0.036	8.7160 ± 0.966	3.6210 ± 0.995	0.9816
*L. acidophilus*	IRPs-F	0.0025 ± 0.006	1.2882 ± 0.013	10.9765 ± 0.590	4.7377 ± 0.600	0.9994
*L. acidophilus*	IRPs-J	0.0007 ± 0.003	1.0592 ± 0.134	15.4090 ± 2.230	1.9021 ± 0.190	0.9872
*L. casei*	Blank	0.0036 ± 0.003	0.3250 ± 0.003	13.2420 ± 0.137	5.2783 ± 0.411	0.9991
*L. casei*	Glc	0.0024 ± 0.004	1.3727 ± 0.033	11.1563 ± 0.411	7.7685 ± 1.439	0.9951
*L. casei*	FOS	0.0014 ± 0.003	1.5581 ± 0.100	16.3880 ± 1.189	3.5369 ± 0.576	0.9893
*L. casei*	IRPs-D	−0.0009 ± 0.005	0.8652 ± 0.108	11.5463 ± 1.167	3.3535 ± 0.607	0.9781
*L. casei*	IRPs-C	0.0010 ± 0.001	0.6834 ± 0.062	14.4619 ± 2.374	1.5738 ± 0.173	0.9959
*L. casei*	IRPs-F	0.0015 ± 0.003	1.3780 ± 0.057	13.6088 ± 1.095	3.6685 ± 0.563	0.9937
*L. casei*	IRPs-J	0.0012 ± 0.0017	1.0089 ± 0.039	16.5403 ± 1.210	3.2632 ± 0.432	0.9989
*M. probiotics*	Blank	0.0092 ± 0.009	0.2972 ± 0.006	10.8403 ± 0.562	3.6337 ± 0.480	0.9969
*M. probiotics*	Glc	0.0047 ± 0.007	1.4475 ± 0.037	12.2159 ± 0.433	5.2508 ± 0.447	0.9958
*M. probiotics*	FOS	0.0032 ± 0.002	1.3239 ± 0.017	13.9015 ± 0.217	4.5839 ± 0.255	0.9989
*M. probiotics*	IRPs-D	0.0032 ± 0.004	0.5493 ± 0.046	11.7971 ± 1.108	3.7584 ± 0.518	0.9662
*M. probiotics*	IRPs-C	0.0010 ± 0.002	0.5791 ± 0.070	12.3725 ± 2.759	1.8580 ± 0.467	0.9695
*M. probiotics*	IRPs-F	0.0016 ± 0.003	0.6356 ± 0.029	9.1068 ± 0.630	3.1537 ± 0.302	0.9942
*M. probiotics*	IRPs-J	0.0008 ± 0.001	0.6635 ± 0.038	12.5165 ± 0.652	3.6694 ± 0.513	0.9899

Among all carbon sources, Glc and FOS significantly promoted growth, with steep curves indicating rapid bacterial proliferation. Of the four IRPs, IRPs-F and IRPs-D had the most positive effects, resulting in faster growth, while IRPs-C and IRPs-J showed weaker effects, with more gradual growth curves. In the MRS medium without added carbon source, the growth of the four probiotics was minimal. Compared to *L. acidophilus*, *L. casei*, and the *M. probiotics*, *L. plantarum* exhibited negligible growth, indicating that its proliferation is severely limited in the absence of a carbon source. This suggests that *L. plantarum* may be more dependent on exogenous carbon sources than the other strains. After 48 h, the growth curves of most groups leveled off, indicating a stable bacterial population. *L. plantarum* exhibited the highest growth under Glc and FOS conditions, surpassing the other probiotics. The bacterial growth order was: Glc > FOS > IRPs-F > IRPs-D > IRPs-J > IRPs-C.

Additionally, *L. plantarum* also demonstrated the strongest ability to utilize carbon sources, while *M. probiotics* showed the weakest response. The differences in growth are likely due to the metabolic characteristics and enzyme activities of the individual strains. *L. acidophilus* and *L. casei* showed weaker responses, particularly with IRPs-C and IRPs-J, which resulted in slow growth and low final proliferation. The growth trend of *M. probiotics* resembled that of *L. plantarum,* suggesting a higher proportion of *L. plantarum* or similar strains in the mixed culture. However, the final proliferation of the *M. probiotics* was lower, likely due to the overall effect being diluted by varying metabolic capacities, resource competition, and other factors. In contrast, a single strain may exhibit higher specificity and efficiency in the metabolism and utilization of specific polysaccharides, without competition or inhibition from other strains.

### 3.7 Analysis of SCFAs

The metabolic end product SCFAs not only reflects the proliferation of probiotics, but also reflects their utilization of carbon sources. Table 2 summarizes the accumulation of SCFAs in cultures of four probiotic strains after 48 h of fermentation with different carbon sources. The results revealed that acetic acid was the predominant SCFAs produced in the metabolic process of four probiotics. In contrast, trace amounts of propionic acid, isobutyric acid, and butyric acid were also detected. Compared to the blank control without carbon source, the SCFAs content in the media containing IRPs as a carbon source was significantly higher. This indicates that probiotics can effectively utilize polysaccharides to produce acidic metabolites during fermentation. *L. acidophilus* exhibited the highest acid production capacity. This can be attributed to its excellent tolerance to acidic environments, allowing it to thrive and produce metabolites even under low pH conditions. Furthermore, during the fermentation of *L. plantarum,* the total SCFAs content was highest when IRPs-F was used as the carbon source, reaching 56.64 ± 1.65 mM, which was higher than that achieved with Glc or FOS. In contrast, *L. casei* produced significantly fewer SCFAs, especially propionic and butyric acids, suggesting a weaker ability to utilize polysaccharides. The *M. probiotics* culture produced the least amount of SCFAs, likely due to factors such as strain competition, differences in metabolic pathways, and the complexity of metabolic regulation. These factors may inhibit the metabolic activity of certain strains, leading to reduced acid production overall.

**Table 2:** Short-chain fatty acids profile in liquid cultures of four probiotic strains after fermentation for 48 h with different carbon sources.

Bacteria	Carbon source	AA (mM)	PA (mM)	IBA (mM)	BA (mM)	Total SCFAs
*L. plantarum*	Glc	41.24 ± 0.34^b^	nd	nd	nd	41.24 ± 0.33^c^
	FOS	36.10 ± 0.37^c^	4.36 ± 0.48^b^	nd	nd	43.45 ± 1.04^b^
	IRPs-D	31.15 ± 0.02^d^	3.82 ± 0.08^b^	nd	nd	34.97 ± 0.08^d^
	IRPs-C	20.96 ± 0.10^f^	3.53 ± 0.28^b^	nd	nd	26.41 ± 0.28^f^
	IRPs-F	50.62 ± 1.25^a^	6.01 ± 0.62^a^	nd	nd	56.64 ± 1.65^a^
	IRPs-J	28.28 ± 0.09^e^	4.31 ± 0.93^b^	nd	nd	32.58 ± 0.98^e^
	Blank	11.68 ± 0.35^g^	4.61 ± 0.60^b^	nd	nd	16.28 ± 0.83^g^
*L. casei*	Glc	35.19 ± 0.01^c^	nd	2.60 ± 0.01	nd	37.80 ± 0.01^c^
	FOS	40.64 ± 0.49^b^	nd	2.41 ± 0.44	nd	43.05 ± 0.79^b^
	IRPs-D	28.00 ± 0.81^d^	4.08 ± 0.03	nd	nd	32.09 ± 0.84^d^
	IRPs-C	28.05 ± 0.32^d^	nd	nd	nd	28.05 ± 0.32^e^
	IRPs-F	43.83 ± 0.10^a^	4.19 ± 0.88	nd	nd	48.50 ± 0.21^a^
	IRPs-J	29.42 ± 1.89^d^	nd	nd	nd	29.42 ± 1.89^e^
	Blank	16.59 ± 1.50^e^	nd	nd	nd	16.59 ± 1.50^f^
*L. acidophilus*	Glc	47.30 ± 1.43^d^	4.79 ± 2.16	2.22 ± 0.27^a^	3.63 ± 0.51^a^	57.94 ± 3.05^b^
	FOS	56.15 ± 1.52^a^	nd	3.26 ± 1.28^a^	3.50 ± 0.32^a^	62.92 ± 3.09^a^
	IRPs-D	51.29 ± 0.10^b^	nd	2.35 ± 0.58^a^	3.19 ± 0.47^a^	56.83 ± 0.96^bc^
	IRPs-C	41.77 ± 0.01^e^	nd	2.80 ± 0.73^a^	nd	44.58 ± 0.73^d^
	IRPs-F	49.26 ± 0.02^c^	5.28 ± 1.22	nd	nd	54.48 ± 1.21^bc^
	IRPs-J	47.80 ± 0.57^cd^	nd	2.61 ± 0.02^a^	3.37 ± 0.59^a^	53.79 ± 0.51^c^
	Blank	17.25 ± 1.33^f^	nd	nd	nd	17.25 ± 1.33^e^
*M. probiotics*	Glc	21.90 ± 0.89^c^	6.48 ± 0.01^a^	nd	3.71 ± 0.13	28.39 ± 0.91^c^
	FOS	35.61 ± 0.41^a^	nd	nd	nd	35.61 ± 0.41^a^
	IRPs-D	27.74 ± 2.56^b^	3.83 ± 0.41^b^	nd	nd	31.57 ± 2.96^b^
	IRPs-C	20.89 ± 0.37^c^	3.95 ± 0.11^b^	nd	nd	24.83 ± 0.11^d^
	IRPs-F	28.84 ± 0.21^b^	nd	nd	nd	28.84 ± 0.21^c^
	IRPs-J	29.46 ± 0.61^b^	nd	nd	nd	29.46 ± 0.61^bc^
	Blank	13.99 ± 0.10^d^	nd	nd	nd	13.99 ± 0.10^e^

AA, acetic acid; PA, propionic acid; BA, butyric acid; VA, valeric acid.

Blank group: sugar-free MRS basic medium.

All results were expressed as mean ± standard deviation of three independent experiments.

The mean values of different letters in the same column were significantly different for each probiotic strain (*p* < 0.05).

Different IRPs significantly influenced the production of SCFAs by *L. acidophilus,* L. *p*, *L. casei,* and *M.p*. By providing effective carbon sources, IRPs promote SCFAs production, especially acetic acid, which can help maintain probiotic growth and metabolic activity, potentially offering intestinal health benefits. The results of both bacterial proliferation and SCFAs production suggest that IRPs-F exhibited metabolic capacity similar to FOS, making it the most promising prebiotic among the IRPs.

### 3.8 Analysis of pH, lactic acid concentration and total sugar content

**Figure 8:**
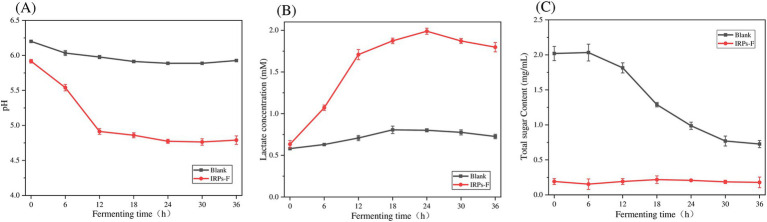
pH value **(A)**, Lactic acid concentration **(B)**, and total sugar content **(C)** of *L. plantarum* after treatment with IRPs-F.

IRPs-F was added to sugar-free MRS medium and inoculated into *L. plantarum* suspension. The change of pH during the growth of *L. plantarum* is shown in Figure 8A. The results showed that the pH value of the experimental group decreased rapidly between 0 and 18 h. between 18 and 24 h, and the pH value decreased slowly. From 24 to 36 h, the pH value was basically stable, and the pH value of the blank group did not change significantly. These findings suggest that when IRPs is used as a carbon source, it can be decomposed and utilized by probiotics, thereby promoting their growth. In this process, SCFA and other metabolites are produced, resulting in a decrease in pH.

During the proliferation of lactic acid bacteria, many acidic metabolites are produced, which play a crucial role in inhibiting harmful bacteria. Therefore, changes in the lactic acid content of the fermentation broth can serve as an indicator of bacterial growth. As shown in Figure 8B, the concentration of lactic acid in the fermentation broth gradually increased with the extension of fermentation time after adding IRPs-F. Initially, the lactic acid concentration increased slowly, but as fermentation progressed, the rate of increase accelerated. When the polysaccharide was fully hydrolyzed and utilized by the bacteria, and the sugar source in the medium was nearly depleted at the end of growth, lactic acid production began to slow and eventually plateau. These results indicate that IRPs-F effectively promoted the growth of *L. plantarum* and significantly enhanced lactic acid production.

As shown in Figure 8C, during the fermentation of *L. plantarum,* the total sugar content in the solution undergoes significant changes. In the initial phase, *L. plantarum* hydrolyzes complex polysaccharides into simple reducing sugars. In the mid-fermentation phase, these hydrolyzed reducing sugars are absorbed by the bacteria and enter the glycolysis pathway, leading to a decrease in the total sugar content of the solution. As fermentation continues, the growth and metabolism of L. *p* gradually stabilize, and the remaining polysaccharides are further hydrolyzed and absorbed, ultimately being converted into metabolites such as lactic acid.

## 4 Discussion

Plant-derived natural medicines have long attracted considerable attention due to their numerous health benefits (Kakar et al., 2023). Among these, plant polysaccharides, as key components of natural medicines, are known for their significant physiological activities, including antibacterial, antioxidant, anti-inflammatory, immune-boosting, and hypoglycemic effects (Yu et al., 2018). For instance, polysaccharides from plants like *Lycium barbarum* and *Ganoderma lucidum* have demonstrated notable anti-tumor and immune-enhancing properties (Wang et al., 2023). Research has shown that polysaccharides from plants such as *Lycium barbarum* not only possess anti-tumor and immune-boosting effects but also promote overall health by enhancing antioxidant defense mechanisms and reducing oxidative stress (Zhu et al., 2022). These bioactive compounds offer a range of therapeutic benefits, reinforcing their potential in health promotion and disease prevention.

Prebiotics are food components that selectively stimulate the growth and activity of beneficial bacteria in the gut (Milani et al., 2017). Common prebiotics include oligosaccharides, FOS, and inulin. In order to play the role of prebiotics, these prebiotics must be able to exist stably in the digestive tract and effectively resist the degradation of gastric acid and digestive enzymes. For polysaccharides, polysaccharides with strong anti-digestibility can maintain stability in the intestine, successfully reach the large intestine and be utilized by the beneficial flora in the intestine, thereby better promoting intestinal health. Human digestive enzymes, such as salivary amylase, pancreatic amylase, and α-amylase, primarily hydrolyze α-glycosidic bonds, while their ability to break down other glycosidic bonds is limited or nonexistent (Wu et al., 2024). For example, FOS, a recognized prebiotic, consists of fructose units linked by β-2,1-glycosidic bonds. These bonds are more chemically stable than α-glycosidic bonds, which endow FOS with strong resistance to enzymatic hydrolysis by α-amylase. Similarly, Fourier transform infrared spectroscopy analysis confirmed the presence of β-glycosidic bonds in all four IRPs, which likely underpins their anti-digestive properties. As a result, the IRPs are not readily digested or absorbed in the upper gastrointestinal tract, allowing them to reach the large intestine for bacterial fermentation and utilization. Consequently, the four IRPs exhibit significant resistance to α-amylase hydrolysis and preliminarily fulfill the anti-digestive standards required for prebiotics.

Polysaccharides play a significant role in regulating the intestinal microbiota and promoting gut health. Numerous studies have demonstrated that polysaccharides exhibit potential prebiotic effects (Zou et al., 2022; Ke et al., 2023). Plant-derived polysaccharides can be utilized by specific probiotics in the intestine. By promoting the growth of beneficial bacteria, they indirectly enhance the structure and function of the gut microbiota and strengthen intestinal barrier function, thereby contributing to overall health. Certain plant polysaccharides stimulate the growth of lactic acid bacteria like *Lactobacillus lactis* and *Lactobacillus plantarum*, which in turn increase the production of metabolites such as SCFAs. These metabolites play a key role in maintaining intestinal pH, inhibiting harmful bacteria, and enhancing immune function (Morrison and Preston, 2016). However, the experimental results show that the proliferative effect of IRPs was significantly reduced in the mixed probiotic group compared to the individual probiotics. This may be due to higher specificity and efficiency in the metabolism and utilization of specific polysaccharides by single strains, with no resource competition or inhibition from other strains. In contrast, the *mixed probiotics* group may experience more dispersed effects due to varying metabolic capacities, resource competition, and other factors.

Beyond improving the gut microbiota, prebiotics also bolster the body’s defense against pathogenic microorganisms through interactions with the intestinal immune system (Shokryazdan et al., 2017). Prebiotics help reduce the risk of intestinal-related diseases by promoting the secretion of immune factors from intestinal epithelial cells, activating local immune responses, and inhibiting intestinal inflammation. Studies have shown that there is a close bidirectional regulatory relationship between the intestinal microbiota and the immune system (Sittipo et al., 2018). A healthy microbiota structure can enhance immune function and improve the body’s resistance to pathogenic microorganisms. Plant polysaccharides not only improve the intestinal microecological environment but also enhance the body’s immune response through their immunomodulatory effects, particularly in fighting infections and inflammation. Therefore, as natural immunomodulators, plant polysaccharides show great promise and can provide strong support for the prevention and treatment of various immune-related diseases by promoting gut microbiota balance and boosting immune responses.

In this paper, as a representative component of natural dietary fiber, polysaccharides extracted from *Imperatae Rhizoma* have been shown to significantly promote the proliferation of probiotics. However, research on their specific mechanisms of action, active ingredients, and processing methods is still in the preliminary stages. Most existing studies focus on *in vitro* experiments, with a lack of clear animal or human trial data. The biological activity of these polysaccharides may be influenced by their complex molecular structure as well as the extraction and purification processes. Additionally, the large-scale extraction technology for IRPs is not yet fully developed, and the high production costs limit their widespread application. Firstly, the extraction process may be influenced by factors such as the source of raw materials, extraction methods, and associated costs. In large-scale production, ensuring consistent quality and an efficient extraction rate is particularly crucial. Secondly, the biological activity of the polysaccharides may be affected by factors like heat, pH, and processing time. Therefore, optimizing extraction and processing technologies is essential to preserve their functionality. Lastly, the compatibility and stability of IRPs in different food matrices need further investigation to ensure their long-term effectiveness in functional foods and to enhance consumer acceptance.

Despite these challenges, as a prebiotic, IRPs have promising prospects in the functional food sector. They can be utilized in dairy products, beverages, and dietary supplements to promote gut health, and are expected to serve as adjuvants in the treatment of intestinal diseases such as irritable bowel syndrome and inflammatory bowel disease when combined with specific probiotic strains. In the future, personalized nutrition programs based on the structural characteristics of IRPs could be developed to optimize gut microbiota, while omics technologies such as metabolomics and genomics can be employed to further explore the regulatory mechanisms on the intestinal flora, providing a scientific foundation for the development of innovative gut health products. As a promising prebiotic resource, IRPs not only significantly support the proliferation of probiotics but also pave the way for the development of novel intestinal health products.

## 5 Conclusion

In this study, the anti-digestibility properties of four different IRPs and their prebiotic activity against four probiotics were investigated. UV and infrared spectroscopy confirmed that the IRPs primarily consist of polysaccharide structures. The IRPs exhibited low digestibility, with α-amylase digestibility of less than 2% and human gastric juice digestibility below 5%. Significant differences were observed in the prebiotic potential of these four polysaccharides. When used as carbon sources to replace glucose for *in vitro* fermentation of *L. plantarum*, *L. casei*, *L. acidophilus*, and *M. probiotics*, IRPs-F demonstrated the most significant effect on promoting *L. plantarum* proliferation. IRPs-F extracted from fresh *Imperata Rhizoma* by hot water extraction, it induced higher proliferation levels of the four probiotics and stimulated greater production of SCFAs. Given their high resistance to digestion, ability to promote the growth of beneficial probiotics, and ability to enhance SCFAs production, IRPs are suggested as potential prebiotics.

## Data Availability

The raw data supporting the conclusions of this article will be made available by the authors, without undue reservation.
