# Supplementary material for: Effects of simulated digestion and prebiotics properties of polysaccharides extracted from Imperatae Rhizoma based on different pilot processes
Source: Front Microbiol. 2025 Mar 7;16:1544261. doi: 10.3389/fmicb.2025.1544261 (PMC11925942; doi:10.3389/fmicb.2025.1544261)
Supplement: Supplementary file 1 [file Data_Sheet_1.docx]

Supplementary Material

# Supplementary Figures and Tables


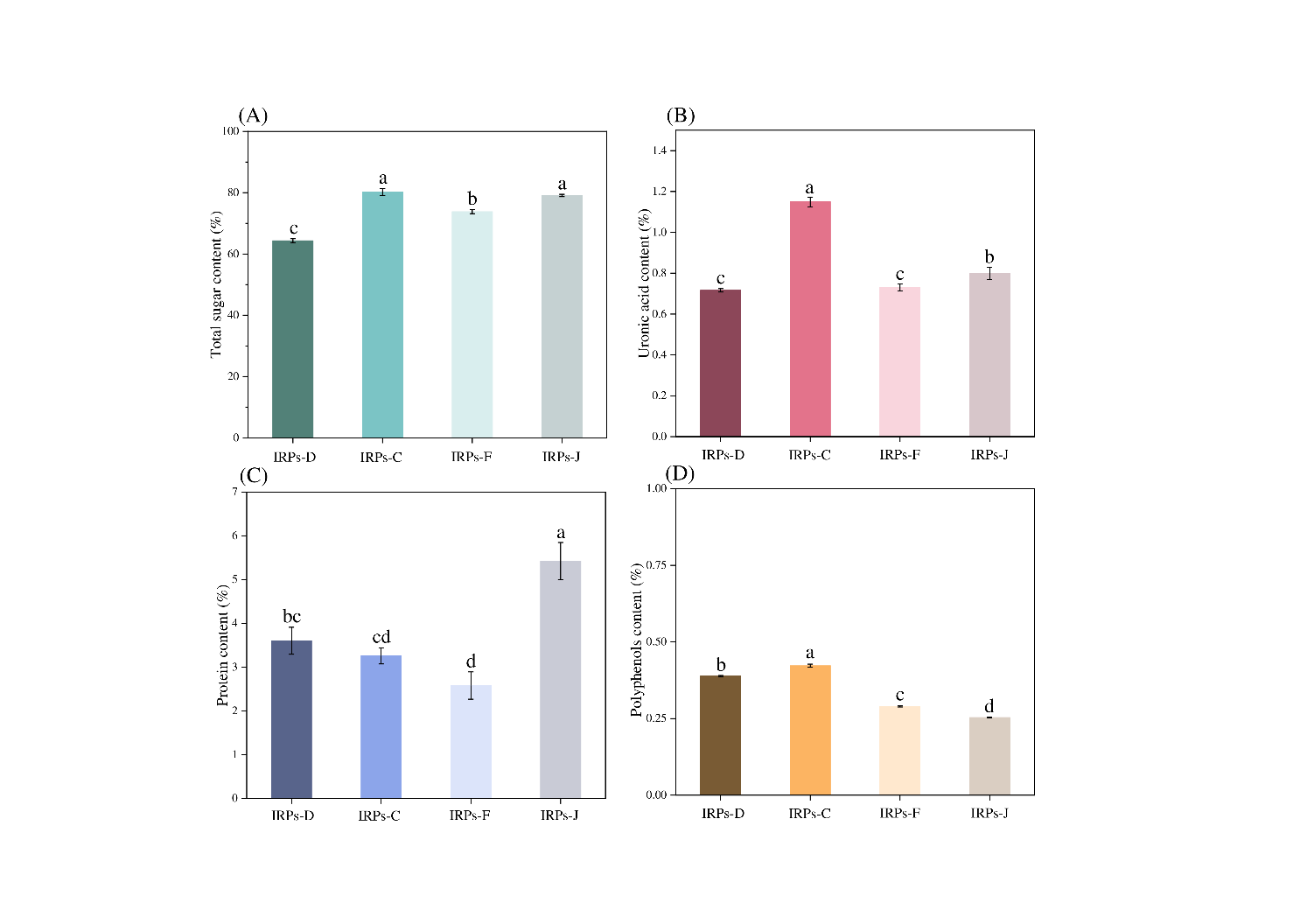


FIGURE 1 The total sugar contents (A), uronic acid content (B), protein content(C), and polyphenols content (D) of four different IRPs. (Different lowercase letters indicate significant differences for p < 0.05).


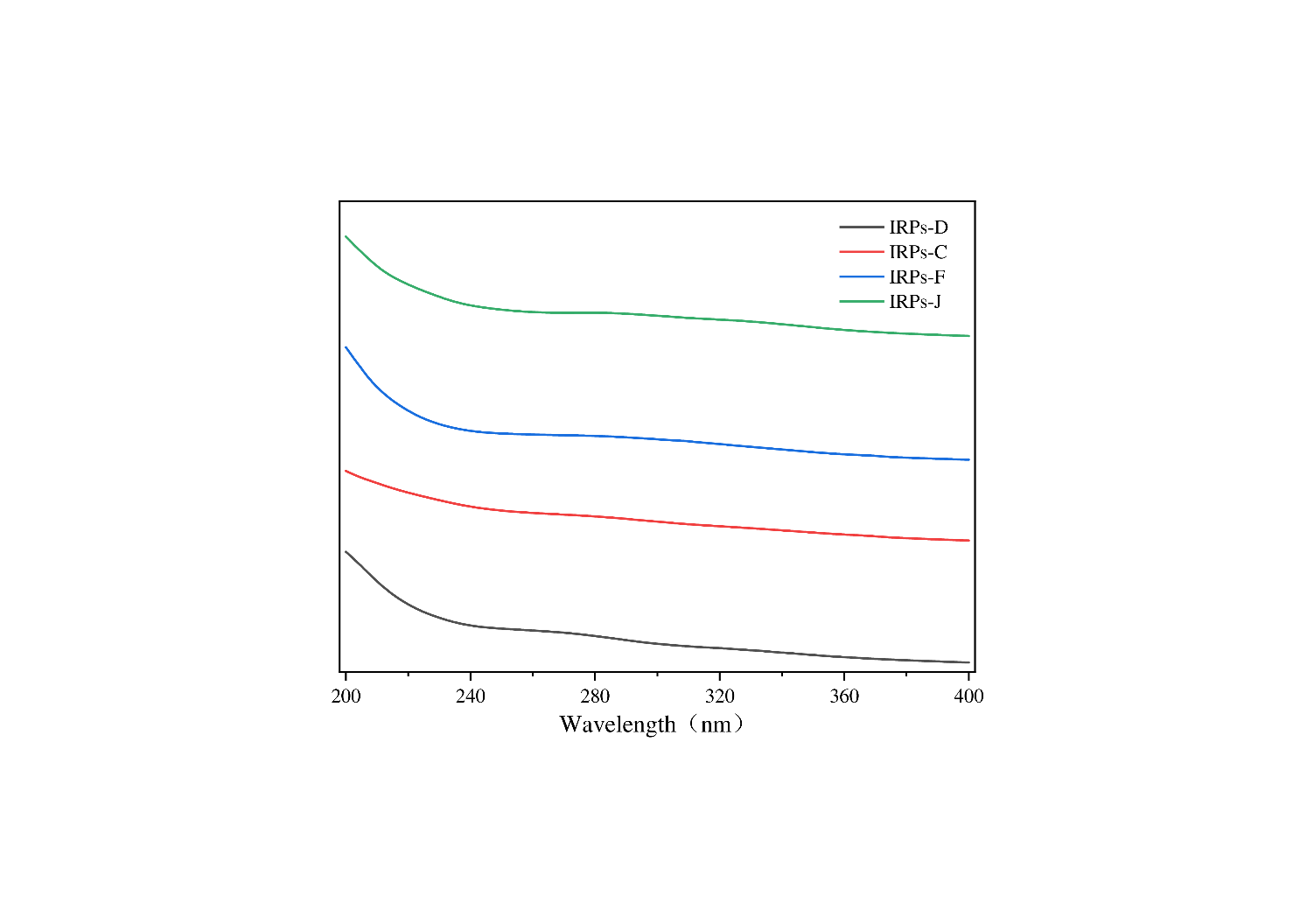
 FIGURE 2 The results of Ultraviolet spectra of different components of IRPs.


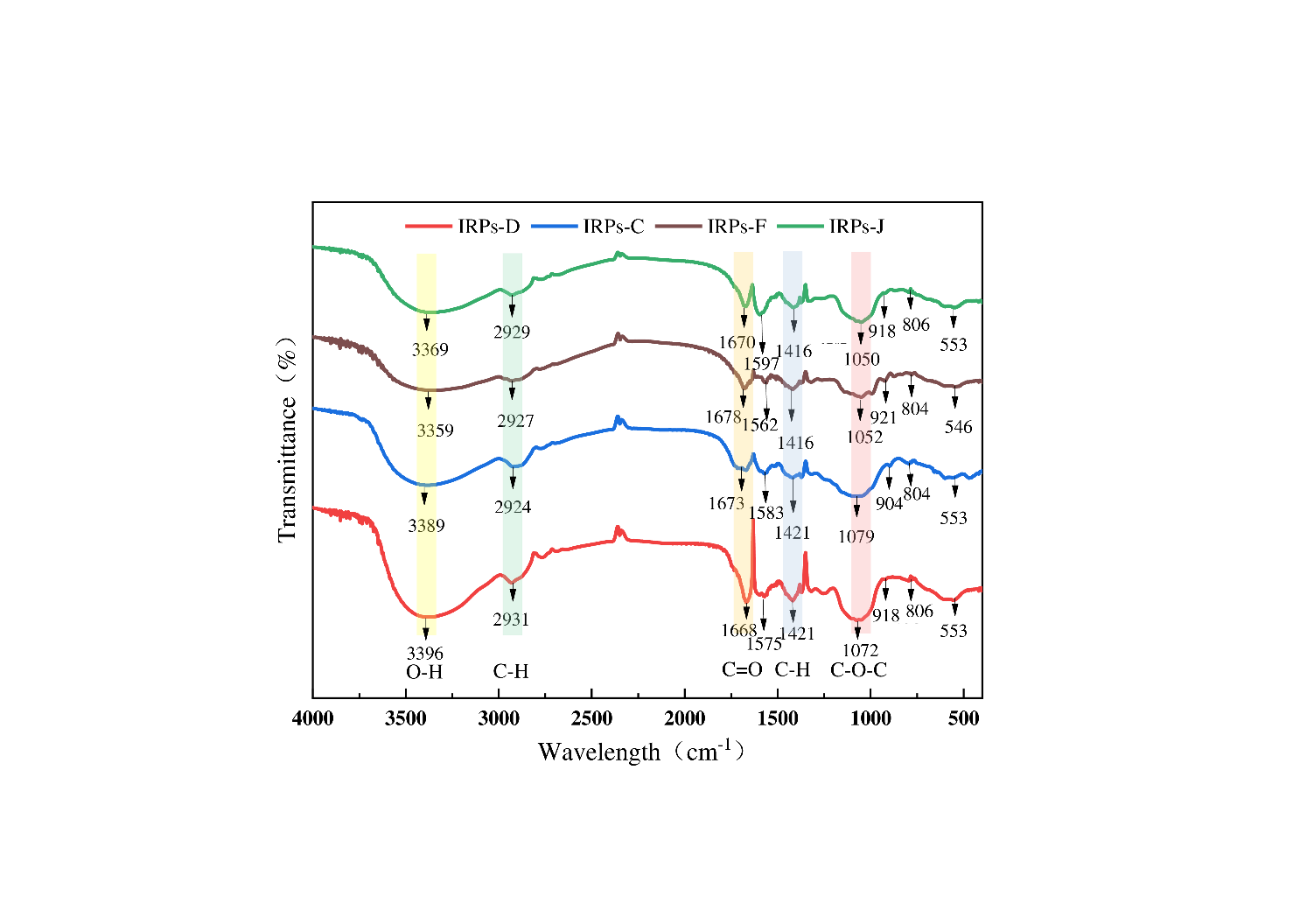
 FIGURE 3 The results of FT-IR spectrograms of four different IRPs.


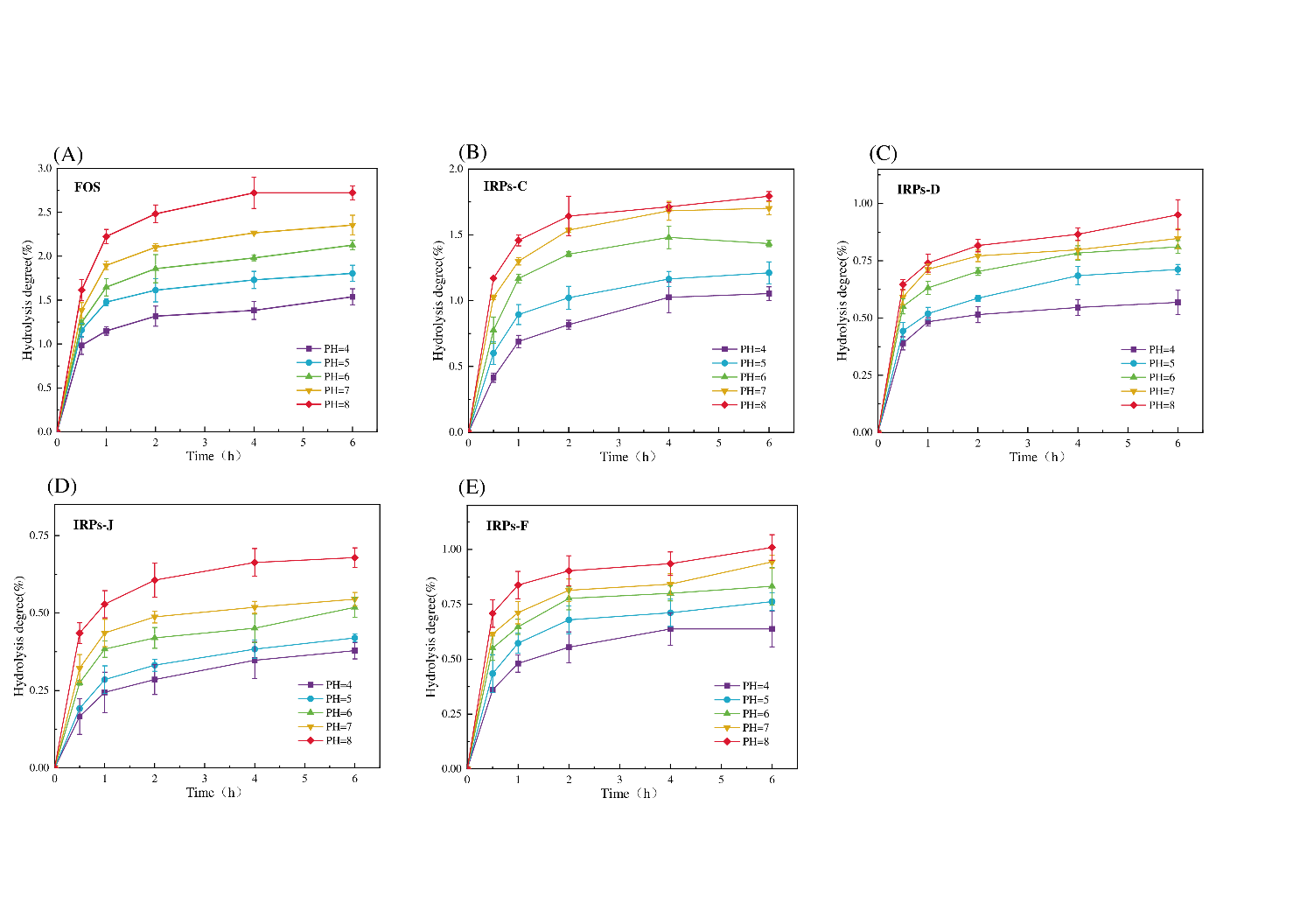
 FIGURE 4 Resistance of FOS and the four IRPs fractions to artificial saliva


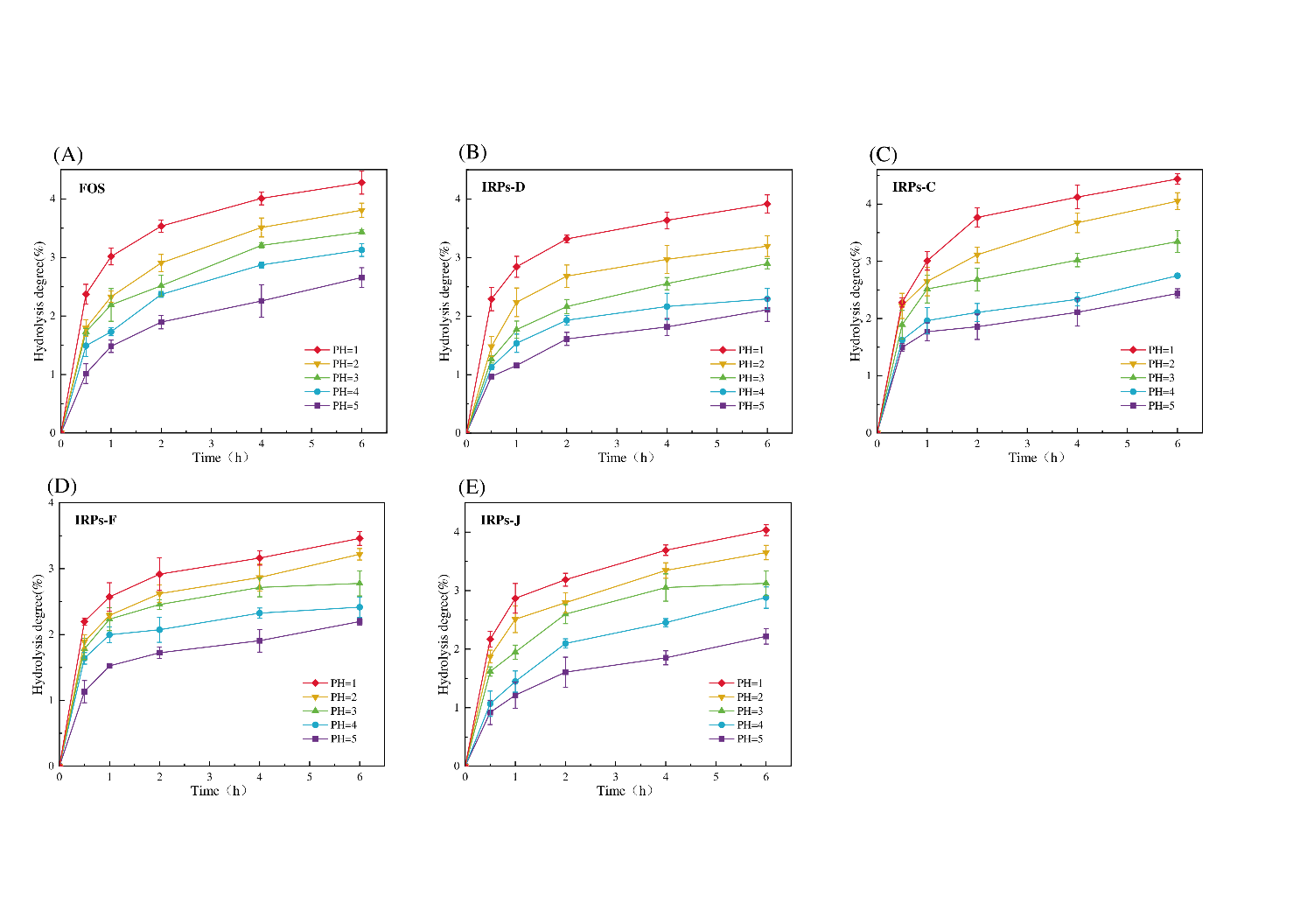
 FIGURE 5 Resistance of FOS and four different IRPs fractions to artificial gastric juice.


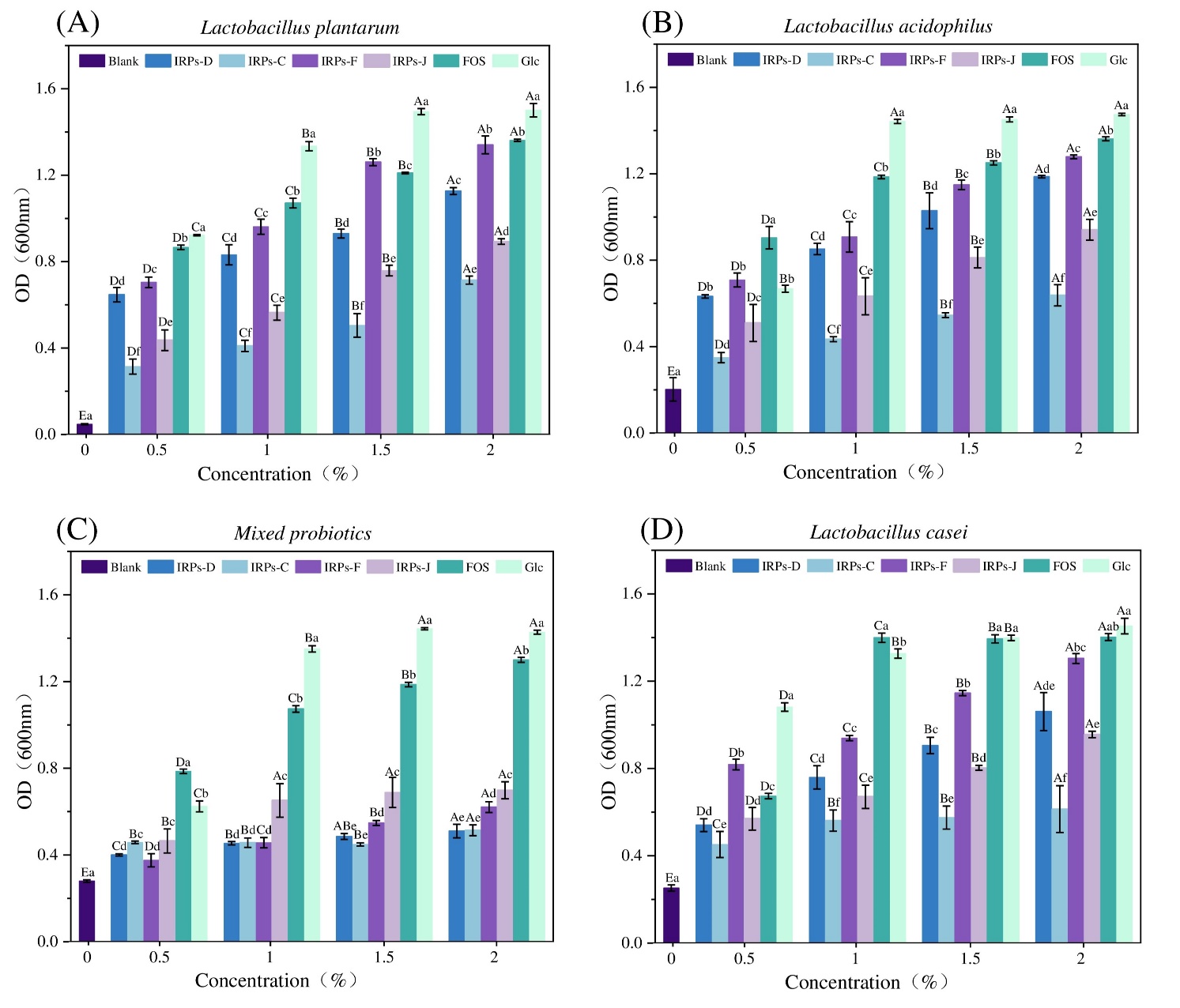
 FIGURE 6 Effects of Glc FOS and four IRPs on the proliferation of Lactobacillus strain after 48 h incubation. *Lactobacillus plantarum* (A), *Lactobacillus acidophilus* (B), *Lactobacillus casei* (C), and *Mixed probiotics* (D).


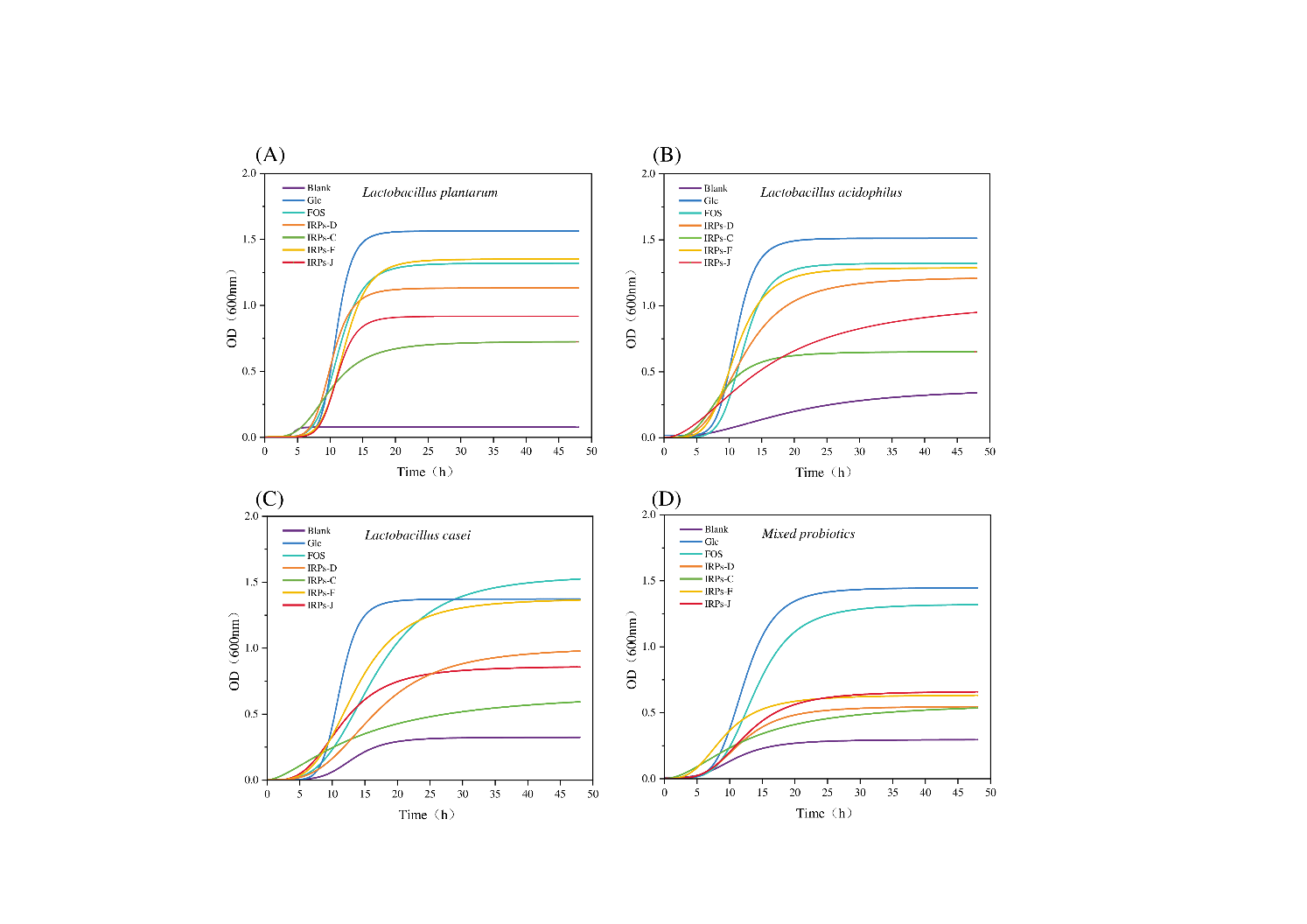
 FIGURE 7 Growth curves of *Lactobacillus plantarum* (A), *Lactobacillus acidophilus* (B), *Lactobacillus casei* (C), and *Mixed probiotics* (D) after treatment with four different IRPs.

Table 1. Growth kinetics equation nonlinear fitting parameters

| Bacteria | Carbon source | A_1_ | A_2_ | X_0_ | p | R^2^ |
| --- | --- | --- | --- | --- | --- | --- |
| *L.plantarum* | Blank | 0.0010±0.008 | 0.0775±0.008 | 4.4392±1.526 | 9.9729±31.696 | 0.8103 |
| *L.plantarum* | Glc | 0.0019±0.003 | 1.5638±0.021 | 10.9427±0.261 | 9.0160±1.053 | 0.9987 |
| *L.plantarum* | FOS | 0.0041±0.006 | 1.3198±0.019 | 11.3484±0.256 | 6.1871±0.578 | 0.9982 |
| *L.plantarum* | IRPs-D | 0.0014±0.003 | 1.1319±0.013 | 10.2045±0.434 | 6.7553±1.306 | 0.9988 |
| *L.plantarum* | IRPs-C | 0.0011±0.001 | 0.7275±0.011 | 10.0886±0.481 | 3.6202±0.403 | 0.9987 |
| *L.plantarum* | IRPs-F | 0.0033±0.004 | 1.3513±0.015 | 12.1811±0.107 | 6.6279±0.389 | 0.9993 |
| *L.plantarum* | IRPs-J | 0.0019±0.005 | 0.9177±0.035 | 11.1006±0.457 | 7.8748±1.586 | 0.9940 |
| *L.acidophilus* | Blank | 0.0026±0.003 | 0.3820±0.088 | 19.3531±4.113 | 2.3102±0.317 | 0.9742 |
| *L.acidophilus* | Glc | 0.0188±0.004 | 1.5120±0.019 | 11.0433±0.283 | 7.2006±0.948 | 0.9986 |
| *L.acidophilus* | FOS | 0.0022±0.003 | 1.3214±0.015 | 12.1154±0.274 | 6.5111±0.478 | 0.9990 |
| *L.acidophilus* | IRPs-D | 0.0021±0.006 | 1.2189±0.031 | 12.0887±0.909 | 3.4356±0.354 | 0.9980 |
| *L.acidophilus* | IRPs-C | 0.0010±0.001 | 0.6533±0.036 | 8.7160±0.966 | 3.6210±0.995 | 0.9816 |
| *L.acidophilus* | IRPs-F | 0.0025±0.006 | 1.2882±0.013 | 10.9765±0.590 | 4.7377±0.600 | 0.9994 |
| *L.acidophilus* | IRPs-J | 0.0007±0.003 | 1.0592±0.134 | 15.4090±2.230 | 1.9021±0.190 | 0.9872 |
| *L.casei* | Blank | 0.0036±0.003 | 0.3250±0.003 | 13.2420±0.137 | 5.2783±0.411 | 0.9991 |
| *L.casei* | Glc | 0.0024±0.004 | 1.3727±0.033 | 11.1563±0.411 | 7.7685±1.439 | 0.9951 |
| *L.casei* | FOS | 0.0014±0.003 | 1.5581±0.100 | 16.3880±1.189 | 3.5369±0.576 | 0.9893 |
| *L.casei* | IRPs-D | -0.0009±0.005 | 0.8652±0.108 | 11.5463±1.167 | 3.3535±0.607 | 0.9781 |
| *L.casei* | IRPs-C | 0.0010±0.001 | 0.6834±0.062 | 14.4619±2.374 | 1.5738±0.173 | 0.9959 |
| *L.casei* | IRPs-F | 0.0015±0.003 | 1.3780±0.057 | 13.6088±1.095 | 3.6685±0.563 | 0.9937 |
| *L.casei* | IRPs-J | 0.0012±0.0017 | 1.0089±0.039 | 16.5403±1.210 | 3.2632±0.432 | 0.9989 |
| *M.probiotics* | Blank | 0.0092±0.009 | 0.2972±0.006 | 10.8403±0.562 | 3.6337±0.480 | 0.9969 |
| *M.probiotics* | Glc | 0.0047±0.007 | 1.4475±0.037 | 12.2159±0.433 | 5.2508±0.447 | 0.9958 |
| *M.probiotics* | FOS | 0.0032±0.002 | 1.3239±0.017 | 13.9015±0.217 | 4.5839±0.255 | 0.9989 |
| *M.probiotics* | IRPs-D | 0.0032±0.004 | 0.5493±0.046 | 11.7971±1.108 | 3.7584±0.518 | 0.9662 |
| *M.probiotics* | IRPs-C | 0.0010±0.002 | 0.5791±0.070 | 12.3725±2.759 | 1.8580±0.467 | 0.9695 |
| *M.probiotics* | IRPs-F | 0.0016±0.003 | 0.6356±0.029 | 9.1068±0.630 | 3.1537±0.302 | 0.9942 |
| *M.probiotics* | IRPs-J | 0.0008±0.001 | 0.6635±0.038 | 12.5165±0.652 | 3.6694±0.513 | 0.9899 |

Table 2. Short-chain fatty acids profile in liquid cultures of four probiotic strains after fermentation for 48 h with different carbon sources.

| Bacteria | Carbon source | AA(mM) | PA(mM) | IBA(mM) | BA(mM) | Total SCFAs |
| --- | --- | --- | --- | --- | --- | --- |
| *L.plantarum* | Glc | 41.24±0.34^b^ | nd | nd | nd | 41.24±0.33^c^ |
|  | FOS | 36.10±0.37^c^ | 4.36±0.48^b^ | nd | nd | 43.45±1.04^b^ |
|  | IRPs-D | 31.15±0.02^d^ | 3.82±0.08^b^ | nd | nd | 34.97±0.08^d^ |
|  | IRPs-C | 20.96±0.10^f^ | 3.53±0.28^b^ | nd | nd | 26.41±0.28^f^ |
|  | IRPs-F | 50.62±1.25^a^ | 6.01±0.62^a^ | nd | nd | 56.64±1.65^a^ |
|  | IRPs-J | 28.28±0.09^e^ | 4.31±0.93^b^ | nd | nd | 32.58±0.98^e^ |
|  | Blank | 11.68±0.35^g^ | 4.61±0.60^b^ | nd | nd | 16.28±0.83^g^ |
| *L.casei* | Glc | 35.19±0.01^c^ | nd | 2.60±0.01 | nd | 37.80±0.01^c^ |
|  | FOS | 40.64±0.49^b^ | nd | 2.41±0.44 | nd | 43.05±0.79^b^ |
|  | IRPs-D | 28.00±0.81^d^ | 4.08±0.03 | nd | nd | 32.09±0.84^d^ |
|  | IRPs-C | 28.05±0.32^d^ | nd | nd | nd | 28.05±0.32^e^ |
|  | IRPs-F | 43.83±0.10^a^ | 4.19±0.88 | nd | nd | 48.50±0.21^a^ |
|  | IRPs-J | 29.42±1.89^d^ | nd | nd | nd | 29.42±1.89^e^ |
|  | Blank | 16.59±1.50^e^ | nd | nd | nd | 16.59±1.50^f^ |
| *L.acidophilus* | Glc | 47.30±1.43^d^ | 4.79±2.16 | 2.22±0.27^a^ | 3.63±0.51^a^ | 57.94±3.05^b^ |
|  | FOS | 56.15±1.52^a^ | nd | 3.26±1.28^a^ | 3.50±0.32^a^ | 62.92±3.09^a^ |
|  | IRPs-D | 51.29±0.10^b^ | nd | 2.35±0.58^a^ | 3.19±0.47^a^ | 56.83±0.96^bc^ |
|  | IRPs-C | 41.77±0.01^e^ | nd | 2.80±0.73^a^ | nd | 44.58±0.73^d^ |
|  | IRPs-F | 49.26±0.02^c^ | 5.28±1.22 | nd | nd | 54.48±1.21^bc^ |
|  | IRPs-J | 47.80±0.57^cd^ | nd | 2.61±0.02^a^ | 3.37±0.59^a^ | 53.79±0.51^c^ |
|  | Blank | 17.25±1.33^f^ | nd | nd | nd | 17.25±1.33^e^ |
| *M.probiotics* | Glc | 21.90±0.89^c^ | 6.48±0.01^a^ | nd | 3.71±0.13 | 28.39±0.91^c^ |
|  | FOS | 35.61±0.41^a^ | nd | nd | nd | 35.61±0.41^a^ |
|  | IRPs-D | 27.74±2.56^b^ | 3.83±0.41^b^ | nd | nd | 31.57±2.96^b^ |
|  | IRPs-C | 20.89±0.37^c^ | 3.95±0.11^b^ | nd | nd | 24.83±0.11^d^ |
|  | IRPs-F | 28.84±0.21^b^ | nd | nd | nd | 28.84±0.21^c^ |
|  | IRPs-J | 29.46±0.61^b^ | nd | nd | nd | 29.46±0.61^bc^ |
|  | Blank | 13.99±0.10^d^ | nd | nd | nd | 13.99±0.10^e^ |

AA: acetic acid, PA: propionic acid, BA: butyric acid, VA: valeric acid,

Blank group: sugar-free MRS basic medium.

All results were expressed as mean ± standard deviation of three independent experiments.

The mean values of different letters in the same column were significantly different for each probiotic strain ( p < 0.05 ).


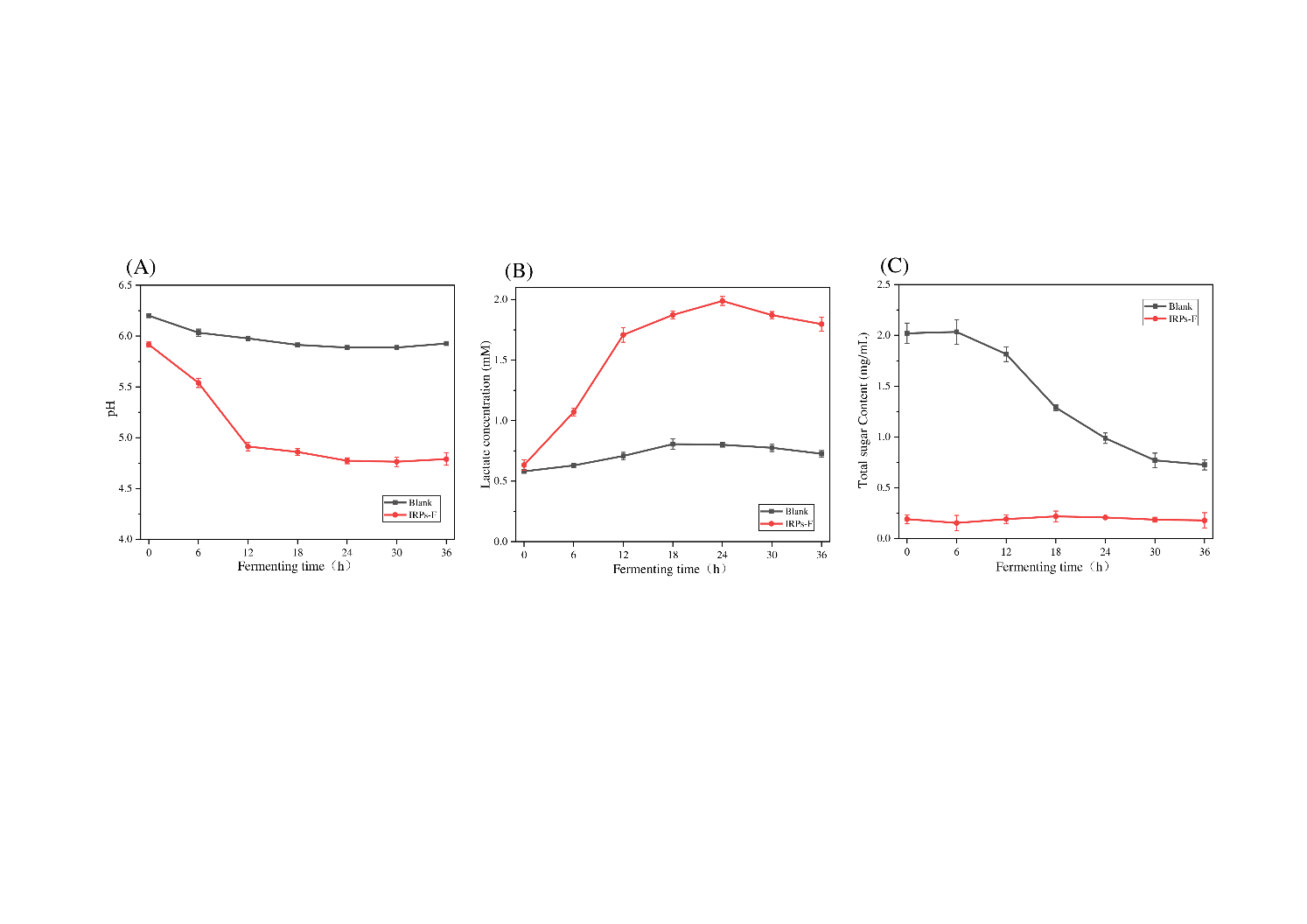
 FIGURE 8 pH value (A), Lactic acid concentration (B), and total sugar content (C) of *L.p* after treatment with IRPs-F.
